# Oxidative stress and expression of insulin signaling proteins in the brain of diabetic rats: Role of *Nigella sativa* oil and antidiabetic drugs

**DOI:** 10.1371/journal.pone.0172429

**Published:** 2017-05-15

**Authors:** Mahmoud Balbaa, Shaymaa A. Abdulmalek, Sofia Khalil

**Affiliations:** Biochemistry Department, Faculty of Science, Alexandria University, Alexandria, Egypt; University of Lancaster, UNITED KINGDOM

## Abstract

**Background and objectives:**

Insulin resistance of the brain is a specific form of type2-diabetes mellitus (T2DM) and the active insulin-signaling pathway plays a neuroprotective role against damaging conditions and Alzheimer’s progression. The present study identifies the mediated emerging effects of the *Nigella sativa* oil (NSO) on the memory enhancing process, its anti-oxidative, acetylcholinestrase (AChE) inhibition, anti-brain insulin resistance and anti-amyloidogenic activities. In addition, the possible role of some anti-diabetic drugs in the neuro-protection processes and their effect in combination with NSO and/or the insulin receptor inhibitor IOMe-AG538 were investigated.

**Methods:**

T2DM-induced rats were orally and daily administrated 2.0 ml NSO, 100 mg metformin (MT), 0.8 mg glimepiride (GI) and different combinations (100 mg MT & 2.0 ml NSO, 0.8 mg GI & 2.0 ml NSO and 2.0 ml NSO & intraperitoneal injection of 1/100 LD50 of IOMe-AG538) per kg body weight for 21 days.

**Results:**

A significant increase in the brain lipid peroxidation and decrease in the antioxidant status with peripheral and central production of pro-inflammatory mediators were observed in diabetes-induced rats. The brain AChE was activated and associated with diminished brain glucose level and cholinergic function. In addition, the brain insulin resistance and the attenuated insulin signaling pathway (p-IRS/ p-AKT/p-GSK-3β) were accompanied by an augmentation in GSK-3β level, which in turn may contribute in the extensive alterations of Tau phosphorylation along with changes in PP2A level. Furthermore, neuronal loss and elevation in Aβ-42 plaque formation were observed due to a low IDE formation and an increased expression of p53, BACE1 and APP with diminished ADAM10, SIRT1 and BDNF levels. The expression profile of AD-related miRNAs in sera and brain tissues displayed its neuro-protection role. The treatment of diabetes-induced rats with NSO and the anti-diabetic drugs alone and/or in combination have the potential to suppress the oxidative stress, the pro-inflammatory mediators and amyloidogenic pathway. Moreover, it lowers the insulin receptor inhibitory effect of IOMe-AG538 and modifies the insulin-signaling pathway. Therefore, it prevents the neurotoxicity, amyloid plaque formation and Tau hyper-phosphorylation and restores AD-related miRNA normal levels.

**Conclusion:**

These data suggest that NSO or its combined treatments with anti-diabetic drugs have a possible benefit as disease modifying agents for the insulin resistance in the brain through enhancing brain insulin signaling pathway.

## Introduction

T2DM and AD shared several pathological features including insulin signaling impairment, oxidative stress and inflammation followed by impaired brain insulin signaling [1]. It was reported that the risk of AD development increased by 45–90% in T2DM [2]. Also, T2DM has a twofold higher risk for AD development when compared to non-diabetic subjects [3]. The brain insulin signaling may be mediated through the activation of phosphorylation of IRS-1 and PI3K/AKT signaling pathway. Consequently, peripheral insulin resistance arises the brain insulin signaling impairment [4] that was described in obese insulin resistance rats [5]. In addition, chronic insulin resistance induces IRS1 phosphorylation at Ser307 and its partial degradation [6]. T2D-associated hyperinsulinemia showed an elevated Aβ accumulation due to a competition between insulin and Aβ for IDE sites and its subsequent inhibition [7]. Also, the hyperglycemia displayed hippocampal damage, elevated p-Tau and GSK3β up-regulation in brain [8]. It is co-existent in brain insulin resistance and deficiency of insulin in AD [3]. Therefore, AD could be faced as brain-specific type of diabetes as type 3 diabetes [3]. Interestingly, AD has insulin and IGF-1 resistance with dysfunctional IRS-1 mediated insulin resistance. The brain insulin resistance may be due to peripheral insulin resistance, reduced insulin uptake by brain and elevated brain Aβ deposition [9]. Several studies investigated the role of insulin resistance and impaired insulin signaling in the AD pathogenesis in brain [10] and neuro-inflammation is mutual interaction between T2DM and AD [11]. Chronic inflammation in T2DM and the leaking of peripheral inflammatory mediators from T2DM to brain induces microglial activation and release of the inflammatory mediators IL-1β, IL-6 and TNF-α in brain [12]. These mediators cause neuronal cell death by elevating the apoptosis rate and decreasing the hippocampal neurogenesis [13]. This activates NF-κB that causes an increase of Aβ levels, prolonged activation of glia cells, cytokines release elevation and reduction in the IDE expression level [14].

Today, the plant-derived medications became an important way to manage the diabetes in the world to avoid the adverse effect linked with conventional hypoglycemic agents [15]. *Nigella sativa* L. is fitting in with Ranunculaceae family and its active components such as thymoquinone, have therapeutic properties like the anti-inflammatory effect for many inflammatory diseases, including encephalomyelitis, colitis, edema, diabetes, neuro-inflammation and joint inflammation [16]. Further, it was confirmed that NSO has a potent anti-oxidant effect [17]. It can prohibit the damage of memory after administration of scopolamine and decrease the AChE action and oxidative stress of the brain in rats [18]. Currently, there are no available therapies for the effective modification of AD and the diagnostic tools now are not able to detect early changes, which take place for years prior to actual symptoms [19]. Searching for biomarkers for AD pathogenesis is the target of scientists all over the world and miRNA is one of these promising biomarkers. miRNAs are short (18–22 nt), single-stranded RNA molecules, that play a role in “fine-tuning” the gene expression by hybridization to specific mRNA and suppressing its effective translation. They are involved in physiological and pathological processes [20]. Specific miRNAs are expressed in the CNS, where they regulate neuronal differentiation, synaptic plasticity and neurite outgrowth [21].

We have previously demonstrated that *Nigella sativa* modifies the changed hepatic insulin receptor signaling in STZ-induced diabetic rats fed with a high-fat diet [22]. The present work aimed to highlight the possible therapeutic effects of NSO and its constituents on induced neurotoxicity. It will shed light on the promising use of tissue and circulating miRNAs profiles as biomarkers in the diagnosis, monitoring of disease progression and therapeutic response in T2D model of induced brain insulin resistance, along with other biomarkers. This will potentially provide inexpensive and comprehensive tools for early diagnosis of AD in T2DM.

## Materials and methods

### Materials

Maxime RT Premix Kit was purchased from INtRon Biotechnology (Catalog # 25082, Korea). DNA Ladder (Catalog # P1473), DEPC-treated water (clear colorless liquid) (Catalog #D5758), Triton^™^X100 (liquid colorless to light yellow, CAS #9002-93-1), STZ (white to yellow powder, CAS #18883-66-4) and the primer sequences (dried in 2 ml screw cap tube) were obtained from Sigma-Aldrich, USA. Antibodies were purchased from Novus Biologicals, USA. MT as Glucophage tablets (500 mg white colored, film-coated tablets) and GI as Amaryl (2 mg light pink colored oval shaped, uncoated tablets) were purchased from Eva Pharma, Egypt. Solvents and other associated biochemical reagents were obtained with high grade from Sigma-Aldrich, USA.

### Preparation of NSO

NSO was prepared as reported from our previous work [22]. The GC-MS analysis of NSO contained thymoquinone, the most active constituent with molecular weight 164, anethole, *p*-cymene, limonene, carvone and ascorbic acid. The oil contained also four saturated and four unsaturated fatty acids [23].

### Rat model of T2DM

Male Wistar albino rats (Rattus norvegicus, n = 180 and BW is 125 ± 20 g) were obtained from the animal house of the Medical Technology Center, Alexandria University. Rats were housed before starting the experiment for 15 days to be adapted to the environment in polycarbonate cages (8 rats/group) in a room with a day-night cycle of 12 hours, temperature of 22±2.0 and humidity of 45% to 46% with a 12 h photoperiod. Food and water were supplied *ad libitum*. All the experimental procedures were run according to the animal protocols approved by the Ethics Committee of Faculty of Science, Alexandria University, Egypt. The rats were fed with a standard balanced commercial diet. To induce T2DM, rats were fed a high-fat diet for 2 weeks (58% fat, 17% carbohydrate and, 25% protein, 310 g/kg butter, 253 g/kg casein, 10 g/kg cholesterol, 60 g/kg vitamins and minerals, 1.0 g/kg yeast powder and 1.0 g/kg sodium chloride). It was freshly prepared per week and stored at 4°C. NPD (365 g/kg) contained 11% fat, 15% carbohydrate and 16% protein [24], which resulted in significant increase in glucose, cholesterol and triacylglycerol levels and the body weights compared to zero day before feeding HFD. On day 14 from HFD, a single intraperitoneal injection of STZ (35 mg/kg) dissolved in 100 mM M sodium citrate buffer, pH 4.5 were given to the overnight fasted rats [25]. The STZ-injected rats showed a significant reduction in body weights of HFD/STZ rats but their weights still higher than zero day before HFD manipulation. Rats had free access to water and food after STZ injection, and in the first 24 hours, 5% glucose was given in their drinking water to counter any initial hypoglycemia. After 72 hours of STZ injection, diabetes was confirmed and the rats with blood glucose level higher than 250 mg/dl were deemed diabetic. Before grouping, out of 90 rats subjected for diabetes induction, 7 rats were died and three rats were eliminated due to sub-diabetic condition (108 mg/dl, 112 mg/dl and 122 mg/dl). Both anthropometric measurement and blood sampling were monitored 4 times during the experimental period (at zero day, after 2 weeks from dietary manipulation, after 3 days from STZ injection and after 12 days from starting the treatment). The levels of blood glucose, TC and TG were determined to sure that diabetes was induced and follow the effect of treatment after 12 days. Rats were subjected to all treatments after confirming diabetes incidence and the treatment endured for 21 days. Diabetic rats treated for 21 days with NSO, MT, GI and combination therapy showed an increase in their body weight compared to untreated rats, whereas a decline of body weight was noticed in all induced groups compared to control group.

### Experimental design and animal treatment

Rats were divided into 3 major groups. Group1: served as control groups (non-diabetic) and subdivided into eight groups; mock-treated, DMSO (2.0 ml/kg), NSO (2.0 ml/kg BW), MT (100 mg/kg BW), GI (0.8 mg/kg BW), NSO-MT (2.0 m/kg—100 mg/kg BW) and NSO-GI (2.0 ml/kg-0.8mg/kg BW). All doses administrated orally by intra-gastric tube for 21 days post diabetes development and NSO-IOMe (oral 2.0 ml/kg BW- i.p.1.0 mg/ml in PBS). Group2: served as induction groups (un-treated) and sub divided into three induced groups; diabetic (D) groups that received HFD for 2 weeks followed by single i.p. injection of STZ (35 mg/kg BW), insulin receptor inhibitor that received IOMe (i.p.1.0 mg / ml in PBS), and D-IOMe-injected. IOMe was prepared as stock as (10 mg/ ml DMSO) and stored at -20°C and injected with a dose of 1/100 LD50 [22]. Group3: served as induced rats treatment groups and subdivided into six groups; NSO, MT, GI, NSO-MT, NSO-GI and NSO- IOMe. At the end of experiment, rats were deprived of food overnight and terminally anaesthetized with sodium pentobarbital (100 mg/kg i.p.) to minimize animal distress and suffering. The brain tissues were rapidly excised, washed with ice cold 0.9% NaCl and dissected into different lobes. Hippocampus were frozen in liquid nitrogen and stored at −80°C for subsequent analyses. The plasma and serum were prepared for different assays.

### Serum biochemical assays

The assays of PGL and lipid profile parameters were determined as recently reported [22]. For oxidant and antioxidant assays, the brain samples were homogenized in 10 ml cold phosphate buffer saline, pH 7.4 per g tissue. All the samples were centrifuged at 6000 rpm for 15 min at 4°C and the total protein levels were determined by the colorimetric assay [26]. The oxidative stress parameters such as TBARS, NO and GSH levels and the activities of XO, SOD, GPx and GST were studied in the brain tissues of all rat groups. Briefly, the lipid peroxidation assays were carried out according to previous method and expressed as TBARS concentration nmol/mg protein [27]. The NO assay was carried out according to preceding description and was expressed as μM/mg protein [28]. The non-enzymatic antioxidant GSH was performed as the previous method and expressed as mg/mg sample protein content [29]. GST was assayed according to preceding method and the activity of GST was expressed as μmol/min/mg protein [30]. The GPx was assayed and the activity of GPx was expressed as nM/min/mg protein [31]. The SOD assay was carried out according to former assay and the activity of SOD was expressed as μg/min/mg protein [32]. The XO was assayed and the activity of XO was expressed as μmol/h/mg protein [33]. Finally, the brain AChE activity was carried as previously described and the obtained values were expressed as μmol/min/mg protein [34].

### Preparation of brain lysate for neuro-inflammation cytokines profile

Frozen brain tissues were homogenized in lysis buffer (150 mM NaCl, 1% Triton X-100 and 10 mM Tris, pH 7.4) containing protease inhibitor, The homogenate was centrifuged at 10,000 g for 10 min at 4°C and the supernatant was collected for measurement of ELISA parameters. Aβ-42, IDE, TNF-α, IL-6, IL-1β, iNOS and AGEs in brain tissues and sera samples were determined by using commercially available kits. All analyses were performed according to the manufacture guidelines. The assay of sera and brain AGEs (Rat AGE) ELISA kit, Cat# STA-817-5, Cell Biolabs, USA), brain Aβ-42 (Rat Aβ1–42 and Aβ1–42 (ELISA kit Cat# E0946r, EIAab, China), sera and brain TNF-α (Rat TNF-alpha ELISA Kit Cat # K0331196, KOMA BIOTECH, Korea), sera and brain IL-6 (Rat IL-6 ELISA Kit Cat# IB39555, Biocompare, USA), brain iNOS (Rat inducible nitric oxide synthase, iNOS ELISA Kit Cat# E0837r, EIAab, China), brain IL-1β (Rat IL-1β ELISA Kit Catalog #: ELR-IL1b, RayBiotech, Norcross GA, US) and brain IDE (Rat IDE ELISA Kit Catalog # RI0339, ABclonal, USA) were performed according the manufacturer's protocols.

### Isolation of RNA

Total RNA was extracted from frozen brain hippocampus tissues using easy-RED^™^ total RNA extraction kit. Briefly, 10–100 mg of brain was homogenized in 250 μl PBS, DEPC-treated H2O and 750 μl Easy-RED^™^ solution was mixed with the homogenate and processed according to manufacture guidelines. RNA concentration was determined by measuring the absorbance at 260 nm and Purity of the RNA preparation was estimated according to the ratio of absorbance readings 260/280.

### Semi-quantitative RT-PCR

Using one-step RT-PCR (Maxime RT/PCR PreMix kit, INtRON) reaction, the cDNA was synthesized and used for amplification of target gene(s) using specific primer sets as shown in Table 1. Briefly, template RNA (below 500ng) and specific primer (10–20 pmol) were added into the Maxime RT/PCR Premix tubes that contains all the components necessary for cDNA synthesis and amplification. The cDNA synthesis reaction was performed at 45°C for 30 min and then 5 min at 94°C for RTase inactivation. The primers then subjected to PCR cycles, each cycle consisting of denaturation at 94°C for 20–60 sec, annealing at 45–68°C for 20–60 sec, extension at 72°C for 1 min and final extension at 72°C for 5 min. Programs are given as denaturation temperature / times / annealing temperature / times / extension temperature / times / number of cycles (Table 1). RT-PCR products were separated on agarose gel and visualized with UV transilluminator box and the gel bands were quantified by using UVIBAND Image quantification software [35].

**Table 1:** Primer sequences and products size of target genes in expected PCR products for semi-quantitative RT-PCR.

Gene	Primer sequence	PCR program	Size (bp)
**APP**	F- 5′-AGAGGTCTACCCTGAACTGC-3′	94/30/55/30/72/60/35	154
	R- 5′-ATCGCTTACAAACTCACCAAC-3′		
**BACE1**	F: 5′-CGGGAGTGGTATTATGAAGTG-3′	94/30/60/30/72/60/30	320
	R: 5′-AGGATGGTGATGCGGAAG-3′		
**RAGE**	F- 5′-CTGGATGCTAGTCCTCAGTCTG-3′	94/60/58/60/72/60/30	500
	R- 5′-CCTTTGCCATCAGGAATCAGAG-3′		
**BDNF**	F- 5′-ATGGGACTCTGGAGAGCGTGAA-3′	94/30/60/30/72/60/30	574
	R- 5′-CGC CAGCCA ATTCTC TTT TTGC-3′		
**SIRT1**	F: 5′-CTTTGCCTCATCTGCATTTT-3′	94/60/59/60/72/90/30	490
	R: 5′-ATTAGGCCAGCATTTTCTCA-3′		
**ADAM10**	F-5′- GTTAATTCTGCTCCTCTCCTGG-3′	94/30/55/30/72/90/30	703
	R- 5′-TGGATATCTGGGCAATCACAGC-3′		
**NF-κB-p65**	F: 5'-GCGCATCCAGACCAACAATAA-3'	95/30/56/30/72/30/35	424
	R: 5'-GCCGAAGCTGCATGGACACT-3'		
**p53**	F: 5'-ATGGAGGAGTCACAGTCGGATA-3'	94/60/52/60/72/60/30	500
	R: 5'-GACTTCTTGTAGATGGCCATGG-3'		
**β-actin**	F- 5′-GCCATGTACGTAGCCATCCA-3′	94/30/58/30/72/30/35	372
	R- 5′-GAACCGCTCATTGCCGATAG-3′		

### Preparation of total brain extract and western blot

Frozen brain hippocampus tissues were washed with ice-cooled TBS (10 mM Tris HCl and 133 mM NaCl, pH 7.4) and 10% (w/v) and homogenized in lysis buffer (100 mM NaCl, 100 mM EDTA,0.5% Nonidet p-40, 0.5% Na-deoxycholate and 10 mM Tris, pH 7.5) containing protease inhibitors. The homogenates were centrifuged at low speed at 2000 g for 10 min at 4°C and the supernatants were collected for measurement of protein concentrations and Western blot. The primary antibodies of [Tau [p ser356] (NBP1-19906)], [AKT1 (NBP2-01724) & AKT1 [p ser473] (NBP2-35349)], [GSK-3β (MAB2506) & GSK-3β [p Ser9] (NB100-81948)], [IRS1 [p Tyr612] (NBP1-73967)], [PP2A-alpha (MAB1653)] and [β-actin (NB600-501)] immune-blots were performed on the prepared total cell extracts according to the previous described method [36]. The immune-reactive bands were detected by enhanced chemiluminescence (ECL detection kit) and the bands were quantified using UVIBAND Image quantification software.

### miRNA isolation and quantitative real time RT-PCR

Total RNA, including small RNAs, was extracted from frozen hippocampus tissues and biological fluids using miRNeasy Mini miRNA isolation kit (Qiagen). The First-strand cDNA from miRNA was synthesized by using the miScript II RT Kit (Qiagen), finally the detection of mature and noncoding miRNA by real-time PCR using SYBR Green miScript SYPR Green PCR Detection Kit (Qiagen) according to manufacture guidelines. Specific forward primer for **9a:** TAGCACCATCTGAAATCGGTTA, **103:** AGCAGCATTGTACAGGGCTATGA, **34a:** CGGTATCATTTGGCAGTGTCT, **21:** CCCGCTAGCTTATCAGACTGATG, **107:** CAGCATTGTACAGGGCTATCA, **26b:** GATTTCAAGTAATCCAGGATAGGCT, **101:** GGGGTACAGTACTGTGATAA, **9:** GGGTCTTTGGTTATCTAGCT, **181c:** GGTAAGGTGCATCTAGTGCAGATAG, and **U6:** CTTCGGCAGCACATATACTAAAAT. The relative quantity of each miRNA in the brain tissues and sera of rats was normalized to U6 RNA and the relative expression fold change was calculated as described before [37].

### Statistical analyses

All data are expressed as mean ±SE. The statistical analyses were carried out by using the paired sample T-test (SPSS version 16) and the difference was considered statistically significant when P < 0.05.

## Results

The data in all illustrated figures were expressed as mean ± SE (n = 8) and significance (p < 0.05) was indicated as “*” and “#” compared to control and diabetic groups, respectively. The educed results from diabetic rats, which received HFD and injected with STZ manifested the symptoms of T2DM. The obtained data for BW, PGL, serum insulin, lipid profile and other parameters were reported in a recently published study [22]. Hyperglycemia was detected by a significant increase in PGL plateau in diabetic rats (581.31 ± 36.31 mg/dl) compared to control normal group (75.53 ± 1.22 mg/dl), whereas I-OMe-AG538-injected rats showed a significant increase of its level (224.62 ± 4.29 mg/dl) compared to control. The treatment of diabetic rats with MT, GI, NSO—MT and NSO—GI showed significant decrease of PGL (265.23 ± 20.67, 359.94 ± 17.29, 112.46 ± 3.94 and 106.62 ± 3.79 mg/dl, respectively) compared to control. Also, serum insulin concentrations of I-OMe-AG538-injected rats showed a marked significant increase (42.80 ± 1.18 μIU/ml) compared to control rats (11.59 ± 1.76 μIU/ml). NSO-treated group showed 1.3-fold increase in insulin level (127.86 ± 1.27 μIU/ml) compared to the diabetic group (101.59 ± 5.78 μIU/ml). I-OMe-AG538-treated non-diabetic and diabetic groups showed 3.7 & 5.5-fold increases in insulin level compared to normal control. Administration of NSO to I-OMe-AG538 group lead to 7.8-fold decreases in insulin level compared to I-OMe-AG538-injected group. The administration of NSO to I-OMe-AG538-injected diabetic group lead to a non-significant effect on insulin level compared to I-OMe-AG538-injected diabetic group. The treatment of diabetic rats with MT, GI, NSO—MT and NSO—GI leads to a reduction of insulin level (1.9, 1.4, 1.1 and 1.3-fold, respectively) compared to control.

### Brain oxidant and antioxidant related markers

Oxidative stress parameters were represented by measuring TBARS, NO levels and XO activities in all experimental rat groups. A highly significant elevation in TBARS, NO levels and XO activities (5.9, 2.3 and 6.5-fold, respectively) were shown in brain tissue of HFD/STZ-induced rats versus the control group confirming neurotoxicity. The treatment of diabetic rats with NSO, anti-diabetic drugs MT, GI or combination therapy of NSO-MT or NSO-GI restored the oxidative stress markers back to normal level in the HFD/STZ in rat brain as indicated by a high significant reduction in TBARS, NO levels and XO activities versus non-treated diabetic groups. TBARS, NO levels and XO activities showed a highly significant elevation in oxidative stress levels in the brain of non-diabetic and diabetic rats injected with IOMe compared to control groups, whereas the oxidative stress markers TBARS, NO and XO after treatment with NSO showed significant decrease in its levels versus diabetic-untreated rats (Table 2). In contrast, HFD/STZ—induced diabetic rats showed significant decreased brain levels of GSH and the GPx, GST and SOD activities compared to control group (3.3, 14, 4, 3.5- fold decrease, respectively). The treatment of diabetic rats with NSO, anti-diabetic reference drugs MT, GI, NSO-MT and NSO-GI showed significant elevations in antioxidant markers versus diabetic non-treated groups confirming the restoration effect of drugs to keep the antioxidant balance. Experimental rats showed a highly significant decreased in antioxidant parameters in non-diabetic rats injected and diabetic rats injected with IOMe compared to control group, a highly significant increase in brain levels of antioxidant markers (GSH, GPx, GST and SOD) were shown after treatment with NSO versus untreated rats (Table 3).

**Table 2:** Changes in oxidative stress parameters in diabetic rat brain after the course of treatment with NSO, antidiabetic drugs, combination therapy and I-OMeAG538.

Groups	TBARS	XO	NO
**Control**	302 ± 16.50^#^	17.20 ± 2.10^#^	553 ± 10.20^#^
**D**	1789 ± 31.80*	110.79 ± 3.60*	1254 ± 57.40*
**DMSO**	872 ± 8.25*^#^	63.95 ± 3.60*^#^	920 ± 30.90*^#^
**NSO**	424 ± 26.70*^#^	13.47 ± 0.68^#^	693 ± 28.80*^#^
**MT**	491 ± 33.60*^#^	30.30 ± 1.50*^#^	632 ± 31.60*^#^
**NSO-MT**	358 ± 14.50^#^	31.70 ± 1.45*^#^	666 ± 20.40*^#^
**GI**	504 ± 20.00*^#^	39.90 ± 3.80*^#^	839 ± 32.70*^#^
**NSO-GI**	331 ± 11.00^#^	23.16 ± 1.65^#^	628 ± 11.40*^#^
**D-NSO**	586 ± 27.40*^#^	36.60 ± 2.90*^#^	535 ± 17.20^#^
**D-MT**	554 ± 24.40*^#^	47.80 ± 5.80*^#^	690 ± 27.90*^#^
**D-GI**	650 ± 27.00*^#^	40.70 ± 3.57*^#^	592 ± 19.10^#^
**D-NSO-MT**	555 ± 26.80*^#^	38.25 ± 1.90*^#^	617 ± 11.50*^#^
**D-NSO-GI**	492 ± 33.00*^#^	47.80 ± 2.66*^#^	519 ± 7.70^#^
**IOMe**	1089 ± 20.70*^#^	82.50 ± 2.20*^#^	990 ± 18.70*^#^
**IOMe-NSO**	328 ± 23.30^#^	30.30 ± 2.08*^#^	578 ± 18.60^#^
**D-IOMe**	1060 ± 28.00*^#^	95.55 ± 5.00*^#^	927 ± 34.60*^#^
**D-IOMe-NSO**	528 ± 14.30*^#^	54.60 ± 1.25*^#^	690 ± 19.20*^#^

Values are expressed as mean± SE (n = 8). Significance:

*p<0.05 (compared to control) and

^#^p<0.05 (compared to diabetics).

TBARS: mmol/mg protein, XO: (μmol/hr/mg protein, NO: μM/mg protein.

**Table 3:** Antioxidant markers alterations in the brain of diabetic rats and after treatment with NSO, antidiabetic drugs, combination therapy and I-OMeAG538.

Groups	GSH	GPx	GST	SOD
**Control**	3000 ± 20.67^#^	5670 ± 13.30^#^	1880 ± 14.00^#^	8610 ± 28.40^#^
**D**	1175 ± 14.10*	400 ± 6.55*	450 ± 11.00*	2460 ± 12.50*
**DMSO**	1245 ± 16.70*	1370 ± 13.00*^#^	610 ± 9.00*	3230 ± 9.89*^#^
**NSO**	3165 ± 15.98^#^	13800 ± 18.95*^#^	2240 ± 12.00*^#^	10700 ± 25.6*^#^
**MT**	2075 ± 14.90*^#^	6460 ± 17.60^#^	1200 ± 15.50*^#^	5590 ± 14.10*^#^
**NSO-MT**	1850 ± 14.62*^#^	12400 ± 16.60*^#^	1000 ± 8.00*^#^	5110 ± 16.30*^#^
**GI**	1812 ± 12.95*^#^	6960 ± 13.60^#^	1040 ± 6.00*^#^	5730 ± 21.60*^#^
**NSO-GI**	1900 ± 12.67*^#^	6720 ± 12.40^#^	1140 ± 6.20*^#^	6130 ± 16.50*^#^
**D-NSO**	3112 ± 18.10^#^	10910 ± 13.40*^#^	1980 ± 8.00^#^	9020 ± 22.20^#^
**D-MT**	2225 ± 17.25*^#^	3590 ± 11.80*^#^	1190 ± 5.00*^#^	6710 ± 9.20*^#^
**D-GI**	1537 ± 15.53*^#^	6480 ± 10.30^#^	1000 ± 7.00*^#^	4660 ± 15.50*^#^
**D-NSO-MT**	1687 ± 12.26*^#^	7660 ± 9.40*^#^	1097 ± 5.39*^#^	6920 ± 11.00*^#^
**D-NSO-GI**	1337 ± 14.60*^#^	6110 ± 12.90^#^	967 ± 3.40*^#^	4230 ± 12.30*^#^
**IOMe**	1437 ± 13.75*^#^	2160 ± 10.97*^#^	540 ± 7.00*	3460 ± 6.10*^#^
**IOMe-NSO**	2025 ± 15.26*^#^	6800 ± 16.10^#^	9140 ± 8.00*^#^	5750 ± 22.20*^#^
**D-IOMe**	1325 ± 13.65*	1500 ± 11.37*^#^	560 ± 6.00*	3010 ± 14.60*
**D-IOMe-NSO**	1725 ± 14.11*^#^	5320 ± 12.20^#^	958 ± 6.11*^#^	5490 ± 14.00*^#^

Values are expressed as mean± SE (n = 8). Significance:

*p<0.05(compared to control) and

^#^p<0.05 (compared to diabetics).

GSH: μg/mg protein, GPx: nmol/min/mg protein, GST: nmol/min/mg protein, SOD: U/mg protein.

### Neuro-inflammation related mediators

Microglial activation was associated with elevated oxidative stress markers and Aβ deposition. Sera and brain inflammatory levels were examined by measuring TNF-α, IL-6, IL-1β and iNOS levels in all experimental rats with the determination of expression level of NFκ-Bp65. Serum peripheral TNF-α and IL-6 inflammatory cytokines levels increased significantly (8 and 6-fold, respectively) in diabetic rats confirming the induction of central inflammation that directly correlated with induction of NF-κBp65 expression levels. Significantly, diminished serum anti-inflammatory levels were observed in diabetic rats treated with NSO, MT, GI and combination therapy. A consistent significant elevation in serum levels of TNF-α and IL-6 was showed in IOMe-injected and diabetic IOMe-injected rats compared to control groups, whereas the anti-inflammatory effect of NSO ameliorates peripheral inflammatory cytokines elevation and significantly decreases the serum levels of TNF-α and IL-6 (Table 4). Also, the data indicate a highly significant elevation in all neuro-inflammatory markers TNF-α, IL-6, IL-1β and iNOS levels in the brain of diabetic rats versus control group (6.7, 8.9, 8.7 and 7.9-fold, respectively). Additionally, the levels of brain inflammatory cytokines were significantly decreased in diabetic groups treated with NSO, anti-diabetic drugs MT, GI, NSO-MT and NSO-GI and the values were restored to be near control levels. While the levels of inflammatory cytokines (TNF-α, IL-6, IL-1β and iNOS) showed a highly significant elevation in brain of either control or diabetic rats injected with IOMe compared to control group and the treatment of induced groups with NSO displayed a highly significant reduction in all measured brain inflammatory markers in diabetic versus untreated rats (Table 5).

**Table 4:** Serum TNF-α and IL-6 levels in diabetic rats after treatment with NSO, antidiabetic drugs, combination therapy and I-OMeAG538.

Group	TNF-α (pg/ml)	IL-6 (pg/ml)
**Control**	16.60 ± 0.24^#^	100.00 ± 2.11^#^
**D**	134.00 ± 2.40*	610.00 ± 3.00*
**DMSO**	75.90 ± 1.90*	402.00 ± 5.01*^#^
**NSO**	14.00 ± 0.32^#^	85.50 ± 3.10*^#^
**MT**	16.10 ± 0.93^#^	100.10 ± 3.02^#^
**NSO-MT**	15.70 ± 0.47^#^	110.50 ± 3.00^#^
**GI**	23.90 ± 1.29*^#^	182.50 ± 3.00*^#^
**NSO-GI**	19.00 ± 0.27*^#^	162.30 ± 1.90*^#^
**D-NSO**	17.01 ± 0.52^#^	120.70 ± 2.91^#^
**D-MT**	24.43 ± 0.83*^#^	188.10 ± 3.94*^#^
**D-GI**	30.70 ± 1.22*^#^	196.50 ± 3.01*^#^
**D-NSO-MT**	18.90 ± 0.24*^#^	122.50 ±2.63^#^
**D-NSO-GI**	22.60 ± 0.22*^#^	250.40 ± 3.99*^#^
**IOMe**	100.50 ± 2.49*^#^	450.50 ± 3.55*^#^
**IOMe-NSO**	45.80 ± 1.13*^#^	198.50 ± 2.03*^#^
**D-IOMe**	127.90 ± 2.84*	650.30 ± 5.67*
**D-IOMe-NSO**	57.20 ± 1.25*^#^	200.50 ± 3.69*^#^

Values are expressed as mean± SE (n = 8). Significance:

*p<0.05 (compared to control) and

^#^p<0.05 (compared to diabetics).

**Table 5:** Neuro-inflammation cytokines levels (pg/mg protein) in the brain of diabetic rats after treatment with NSO, antidiabetic drugs, combination therapy and I-OMeAG538.

Groups	TNF-α	IL-6	IL-1β	iNOS
**Control**	14.60 ± 0.22^#^	106.62 ± 2.14^#^	11.50 ± 0.25^#^	10.16 ± 0.09^#^
**D**	95.50 ± 1.40*	572.46 ± 3.76*	96.00 ± 0.95*	79.40 ± 0.67*
**DMSO**	40.90 ± 1.10*^#^	314.50 ± 5.04*^#^	41.80 ± 0.33*^#^	38.30 ± 0.33*^#^
**NSO**	14.20 ± 0.36^#^	92.50 ± 3.60^#^	10.30 ± 0.23^#^	9.00 ± 0.19^#^
**MT**	17.10 ± 0.23^#^	128.10 ± 3.62*^#^	16.40 ± 0.23*^#^	11.60 ± 0.19^#^
**NSO-MT**	18.70 ± 0.17^#^	140.50 ± 3.06*^#^	13.50 ± 0.20^#^	10.40 ± 0.17^#^
**GI**	17.90 ± 0.29^#^	152.50 ± 3.72*^#^	19.70 ± 0.44*^#^	12.20 ± 0.14^#^
**NSO-GI**	19.20 ± 0.27*^#^	152.30 ± 1.91*^#^	15.80 ± 0.30^#^	12.20 ± 0.14^#^
**D-NSO**	16.00 ± 0.32^#^	162.70 ± 3.71*^#^	17.90 ± 0.32*^#^	13.40 ± 0.13^#^
**D-MT**	23.40 ± 0.33*^#^	178.10 ± 3.64*^#^	23.22 ± 0.34*^#^	23.90 ± 0.67*^#^
**D-GI**	22.70 ± 0.22*^#^	190.50 ± 3.11*^#^	25.30 ± 0.25*^#^	17.60 ± 0.25*^#^
**D-NSO-MT**	18.30 ± 0.24^#^	222.50 ±3.63*^#^	21.00 ± 0.44*^#^	13.40 ± 0.42^#^
**D-NSO-GI**	24.70 ± 0.22*^#^	240.40 ± 3.03*^#^	22.60 ± 0.50*^#^	21.24 ± 0.48*^#^
**IOMe**	66.50 ± 0.49*^#^	440.50 ± 4.55*	72.00 ± 0.95*^#^	62.10 ± 0.39*
**IOMe-NSO**	22.80 ± 0.13*^#^	216.50 ± 2.43*^#^	24.40 ± 1.00*^#^	13.70 ± 0.40^#^
**D-IOMe**	75.90 ± 0.84*	480.30 ± 5.46*	85.00 ± 0.83*	50.00 ± 0.60*
**D-IOMe-NSO**	37.20 ± 0.25*^#^	228.50 ± 3.68*^#^	25.20 ± 0.66*^#^	14.70 ± 0.39*^#^

Values are expressed as mean± SE (n = 8). Significance:

*p<0.05 (compared to control) and

^#^p<0.05 (compared to diabetics).

### Cholinergic function marker

The activity level of AChE was significantly increased in the brain of diabetes- induced rats versus control (3.3-fold). The brain cholinergic function in diabetic groups treated with NSO, MT, GI and combination therapy were improved significantly by a significant decrease in AChE activity in all treated rats. This decrease was correlated with normalization of brain glucose levels in all rat groups. These observed values are near control levels confirming its neuro-protection role as AChE inhibitor. Also, the rats received IOMe only or diabetic rats injected with IOMe showed a highly significant elevation in AChE activity that was associated with significant diminished in glucose level compared to control groups. Oral administration of NSO to these induced groups showed a significant diminished AChE activity associated with a significant elevation of glucose level in all treated rats (Table 6).

**Table 6:** AChE activities and glucose levels in the brain of diabetic rats and after treatment with NSO, antidiabetic drugs, combination therapy and I-OMeAG538-injection.

Groups	AChE (μmol/min/mg protein)	Glucose (mg/mg protein)
**Control**	93.60 ± 1.34^#^	0.78 ± 0.04^#^
**D**	306 ± 9.60*	0.13 ± 0.02*
**DMSO**	255 ± 6.98*	0.25 ± 0.01*
**NSO**	68.8 ± 5.20*^#^	0.71 ± 0.09^#^
**MT**	116 ± 4.90*^#^	0.59 ± 0.01*^#^
**NSO-MT**	166 ± 5.10*^#^	0.74 ± 0.04^#^
**GI**	110 ± 2.90*^#^	0.44 ± 0.02*^#^
**NSO-GI**	131 ± 1.40*^#^	0.46 ± 0.01*^#^
**D-NSO**	81 ± 3.30*^#^	0.74 ± 0.09^#^
**D-MT**	100 ± 6.60^#^	0.45 ± 0.07*^#^
**D-GI**	146 ± 6.87*^#^	0.52 ± 0.01*^#^
**D-NSO-MT**	122 ± 4.90*^#^	0.50 ± 0.09*^#^
**D-NSO-GI**	123 ± 4.70*^#^	0.49 ± 0.01*^#^
**IOMe**	227 ± 4.30*	0.29 ± 0.05*
**IOMe-NSO**	149 ± 5.20*^#^	0.51 ± 0.03*^#^
**D-IOMe**	265 ± 6.70*	0.19 ± 0.06*
**D-IOMe-NSO**	121 ± 4.80*^#^	0.45 ± 0.09*^#^

Values are expressed as mean± SE (n = 8). Significance:

*p<0.05 (compared to control) and

^#^p<0.05 (compared to diabetics).

### AGEs and brain insulin resistance

The results showed a significant increase in sera and brain AGEs concentrations in diabetic rats compared to control (12.5 and 13-fold, respectively). The treatment of diabetes-induced rats with NSO, anti-diabetic drugs MT, GI, NSO-MT and NSO-GI showed a significant reduction in sera and brain concentrations of AGEs. The control and diabetic rats injected with IOMe showed a highly significant elevation in sera and brain AGEs levels, while the treatment of these induced rats with NSO showed a significant decrease in sera and brain levels of AGEs near to normal values (Table 7).

**Table 7:** Alteration in AGEs levels in brain and in serum of diabetic rats after treatment with NSO, antidiabetic drugs, combination therapy and I-OMeAG538.

Groups	Brain AGEs (U/mg protein)	Serum AGEs (U/ml)
**Control**	0.10 ± 0.01^#^	2.00 ± 0.09^#^
**D**	1.32 ± 0.07*	25.20 ± 0.19*
**DMSO**	0.49 ± 0.06*^#^	11.70 ± 0.1*^#^
**NSO**	0.07 ± 0.01^#^	1.30 ± 0.06*^#^
**MT**	0.082 ± 0.01^#^	2.20 ± 0.04^#^
**NSO-MT**	0.068 ± 0.02^#^	1.80 ± 0.02^#^
**GI**	0.18 ± 0.05^#^	2.10 ± 0.05^#^
**NSO-GI**	0.11 ± 0.03^#^	1.94 ± 0.09^#^
**D-NSO**	0.094 ± 0.01^#^	0.95 ± 0.03*^#^
**D-MT**	1.68 ± 0.04*^#^	3.00 ± 0.04*^#^
**D-GI**	2.26 ± 0.05*^#^	5.60 ± 0.04*^#^
**D-NSO-MT**	0.08 ± 0.01^#^	1.20 ± 0.06*^#^
**D-NSO-GI**	0.13 ± 0.03^#^	3.80 ± 0.07*^#^
**IOMe**	0.79 ± 0.04*^#^	19.20 ± 0.13*
**IOMe-NSO**	0.26 ± 0.03*^#^	3.30 ± 0.09*^#^
**D-IOMe**	1.46 ± 0.06*	28.70 ± 0.17*
**D-IOMe-NSO**	0.30 ± 0.03*^#^	5.60 ± 0.04*^#^

Values are expressed as mean± SE (n = 8). Significance:

*p<0.05 (compared to control) and

^#^p<0.05 (compared to diabetics).

### Regulation of Aβ-42 clearance by IDE

**Fig 1:**
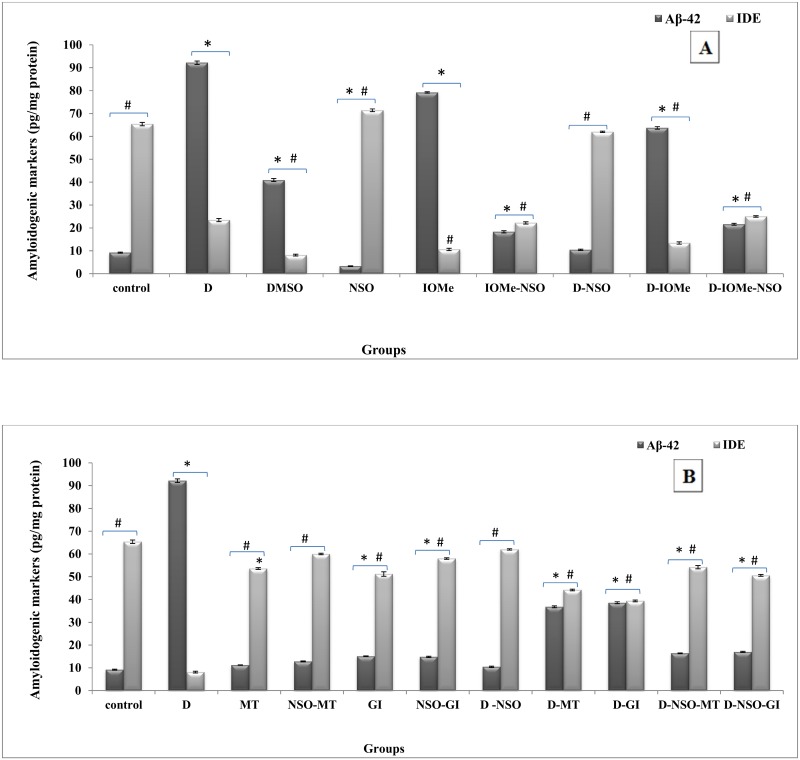
Variations in brain Aβ-42 and IDE content. Aβ-42 & IDE levels in brain of diabetic and I-OMeAG-538-injected rats post NSO treatment (A). Aβ-42 & IDE levels in brain homogenate of diabetic rats after the course of treatment with NSO, reference drugs and combination therapy (B).

A significant elevation of Aβ-42 levels in the brain of diabetic, IOMe-injected and diabetic IOMe-injected rats was noticed (10, 6 and 7-fold, respectively). It is correlated with a significant reduction in IDE levels (8, 6.5 and 5-fold, respectively) (Fig 1A). The treatment of the induced groups with NSO exhibited a marked decrease in Aβ-42 levels with values near to control group associated with a significant restoration of IDE levels (Fig 1A). The treated diabetic rats with NSO, MT, GI and their combination therapy showed a marked inhibition of Aβ-42 deposition in all groups with a significant elevation in brain IDE levels, which clear the Aβ-42 accumulation (Fig 1B).

### Gene expression biomarkers

**Fig 2:**
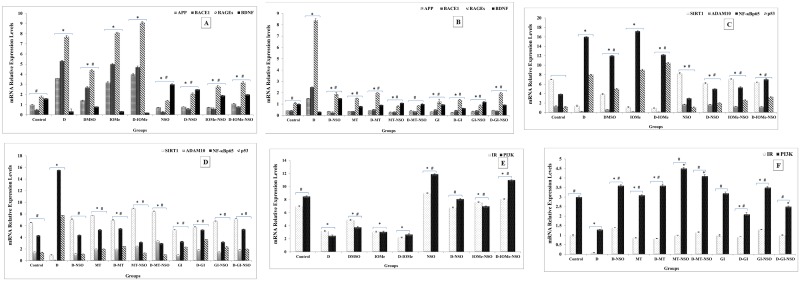
Gene expression profiles of brain amyloidogenic, neuro-protection and insulin signaling biomarkers. mRNA levels by RT-PCR analysis for APP, BACE1, BDNF and RAGEs in brain tissues of diabetic and I-OMeAG538- injected rats versus NSO- treated rats (A). mRNA levels of APP, BACE1, BDNF and RAGEs in the brain of diabetic rats post the treatment with NSO, reference drugs and combination therapy (B). mRNA levels SIRT1, ADAM10, NF-κBp65 and p53 in the brain of diabetic rats, I-OMe-injected rats versus NSO treated rats (C). mRNA levels of SIRT1, ADAM10, NF-κBp65 and p53 in brain tissues of diabetic rats, and after treatment with NSO, reference drugs and combination therapy for (D). mRNA levels of IR and PI3K in the brain of diabetic rats, I-OMeAG538-injected rats versus NSO treated rats (E). mRNA levels of IR and PI3K in the brain of diabetic rats, and after treatment with NSO, MT, GI and combination therapy for IR and PI3K (F). β-actin was used as an internal control and quantification of bands using UVIBAND Image quantification software.

Amyloidgenesis was observed in the diabetes-induced groups received no treatment during the course of induction and IOMe-injected diabetic or non-diabetic rats. They displayed a strong expression of APP, BACE1 and RAGEs in D-induced group, IOMe-injected group and D-IOMe -injected group and linked with very weak expression of BDNF compared to control group (Fig 2A). NSO treatment exhibited a significant diminishment in APP, BACE1 and RAGEs expression levels in D-induced group, in IOMe -injected and in D-IOMe-injected. A significant elevation of BDNF expression level was displayed compared to diabetic groups confirming the discontinuation role of NSO to the amyloidogenic pathway induced by neurotoxicity of STZ-induction and IOMe (Fig 2A). The treatment of NSO, MT, GI and their combination therapy of D-induced groups exerted more protection through marked inhibition in the expression levels of APP, BACE1 and RAGEs and a significant elevation in BDNF expression levels (Fig 2B). On the other hand, HFD/STZ, IOMe-injected rats and D-IOMe-injected group showed a highly significant reduction in SIRT1 expression. In addition, there is a significant depression in ADAM10 levels and a significant induction of p53 and NF-κBp65 levels versus the control group (Fig 2C). The treatment of these induced rats with NSO restored the expression levels of SIRT1 and its signaling parameters ADAM10 with significant depression in p53 and NF-κBp65 expression levels compared with diabetic rats (Fig 2C). NSO, MT and GI treatment alone or in combination with NSO significantly induced respectively the expression of SIRT1, and its signaling markers ADAM10. In addition, a significantly respective depression of p53 and NF-κBp65 expression levels compared with untreated diabetic rats were noticed (Fig 2D). By testing the expression of insulin signaling-related genes, the results showed that HFD/STZ, IOMe-injected and D-IOMe-injected groups showed a highly significant reduction in IR and PI3K expression levels compared to control groups linked with diminished insulin signaling pathway. The treatment of these induced rats with NSO induced the expression of IR and PI3K versus the diabetic rats (Fig 2E). NSO, MT and GI treatment alone or the combination therapy markedly enhanced the expression of IR and PI3K versus the diabetic group (Fig 2F).

### Brain insulin resistance-related biomarkers

**Fig 3:**
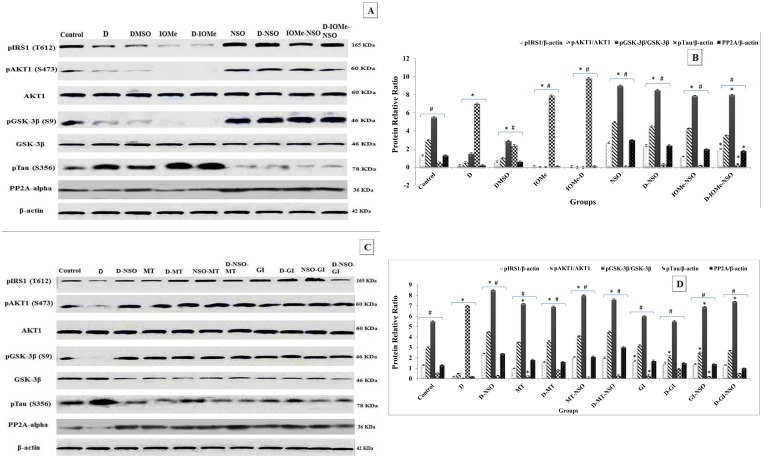
Representative western blotting analyses of brain insulin-induced signaling molecules. Protein level profile of p-IRS1-Tyr612 was normalized to the total amount of β-actin protein and p-AKT1-Ser473 level was normalized to the total amount of AKT1. p-GSK3β-Ser9 was normalized to the total amount of GSK3β. p-Tau-Ser356 and PP2A-alpha levels were normalized to the total amount of β-actin protein in the brain tissue of diabetic rats injected with IOMe and treated with NSO (A). Protein profile of diabetic rats after the treatment with NSO, anti-diabetic drugs and combination therapy is shown as (C). The quantification of protein bands is represented as mean ± SE (n = 5/group) (B) and (D).

Diabetes induced rats, IOMe-injected rats and diabetes- IOMe-injected rats displayed brain insulin resistance. The attenuation of signaling pathway was respectively exhibited by significant inhibition (under-detection) of phosphorylation of signaling proteins (IRS1-pTyr612 (10, 20 and 25-fold), AKT1-pSer473 (6, 15 and 20-fold), GSK-3β-pSer9 (3.6, 25 and 30-fold) compared with control groups) with a significant induction of GSK-3β levels (4, 5 and 10-fold). This was followed by a marked elevation and hyper-phosphorylation of Tau-pSer356 (14, 16 and 20-fold, respectively) that is strongly associated with significant reduction in PP2A levels (4, 4.5 and 3.9-fold, respectively) in brain compared to control group. The treatment of these induced rats with NSO showed a significant activation of brain insulin signaling parameters and marked inhibition of IOMe effect. This was confirmed by a significant respective increase in the protein levels of IRS1-pTyr612, AKT1-pSer473, GSK-3β-pSer9 compared to diabetic group. There is an also a significant decrease in GSK-3β levels linked with marked reduction in Tau-pSer356 levels and an associated increase in its PP2A levels compared to diabetic rats (Fig 3A and 3B). The treated diabetic rats with NSO, anti-diabetic drugs MT and GI and a combination therapy showed a respective enhancement of insulin signaling pathway demonstrated by the proteins IRS1-pTyr612, AKT1-pSer473 and GSK-3β-pSer9 versus diabetic group. These molecules enhanced the subsequent insulin signaling pathway and caused a significant decline in GSK3β protein level that inhibits its kinase activity and Tau-pSer356 and linked with elevated PP2A level (Fig 3C and 3D).

### Modification of brain AD-related miRNA expression profile

**Fig 4:**
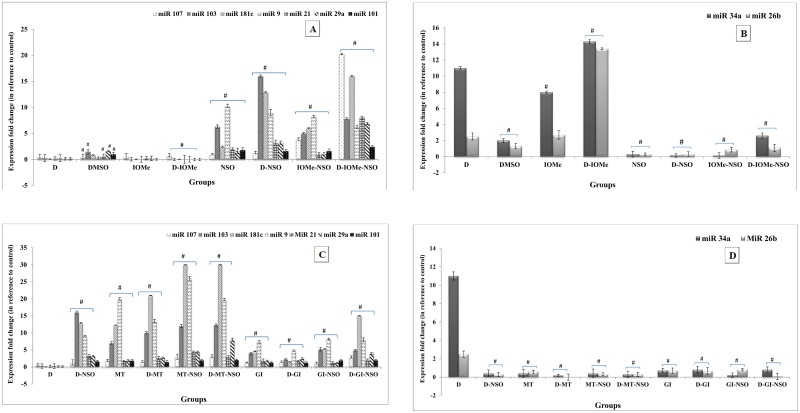
Quantitative RT-PCR analyses of brain AD-related miRNAs expression profile. Alterations in the expression levels of AD-related miRNAs (107, 103, 9, 181c, 21, 101 and 29a) in the brain homogenate of experimental diabetic rats injected with IOMe and treated with NSO (A). Alterations in the expression level of miRNAs 34a and 26b in the brain of IOMe-injected diabetic rats and treated with NSO (B). Alterations in the expression levels of AD-related miRNAs (107, 103, 9, 181c, 21, 101 and 29a) in the brain of diabetic rats after treatment with NSO, anti-diabetic drugs and combination therapy (C). Alterations in the expression level of miRNAs 34a and 26b in the brain of experimental diabetic rats after treatment with NSO, anti-diabetic drugs and combination therapy (D). Significance is shown as (#p < 0.05) compared to diabetes-induced rats.

Alterations in the expression levels of brain AD-related miRNAs (107, 103, 9, 181c, 21, 101 and 29a), which are normally with higher expression in normal brain tissue showed a significant reduction in their expression levels of miRNAs in diabetic, IOMe-injected non-diabetic and diabetic rats. The administration of NSO to these rats showed a significant elevation in the expression levels of all miRNAs in comparison to diabetes-induced rats (Fig 4A). On the other hand, the expression level of miRNAs 34a and 26b (normally with low expression levels in normal brain tissue) were significantly elevated in the brain of diabetes-induced and IOMe-injected rats. The diabetic rats injected with IOMe showed a highly significant elevation in the expression levels in compared to diabetes-induced rats. Administration of NSO alone causes a significant reduction in the expression levels of miRNAs in all rat groups compared with diabetes-induced rats (Fig 4B). In addition, the administration of NSO alone or anti-diabetic drugs MT or GI ameliorate the repression caused by diabetes induction and lead to a significant elevation in the expression of measured miRNAs (107, 103, 9, 181c, 21, 101 and 29a) in comparison to diabetes-induced rats. Moreover, the NSO-MT combination therapy significantly increased the expression of measured miRNAs in brain of diabetic rats in comparison with other treatments (Fig 4C). In contrast, administration of NSO alone, anti-diabetic drugs or combination therapy caused a significant reduction in the expression levels of miRNAs 34a and 26b in comparison to diabetes-induced rats. The treatment with MT significantly decreased the expression of miRNAs 34a and 26b in the brain of diabetic rats in comparison to other treatments (Fig 4D).

### Serum miRNA expression levels in the HFD/STZ-treated rats

**Fig 5:**
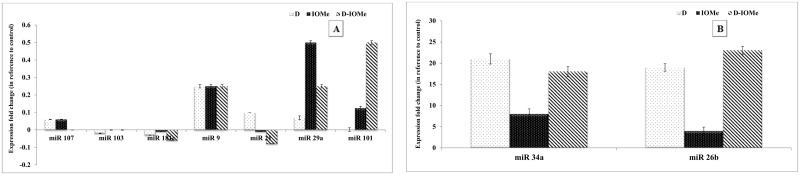
Decreased miRNA expression levels in the blood serum of HFD/STZ-induced rats. The expression levels of miRNAs miR-107, miR-181c, miR-103, miR-101, miR-29a, miR-21 and miR-9 expression levels were down-regulated in the serum of diabetic rats and IOMe-injected rats (A). The expression levels of miRNAs miR-34a and miR-26b were up-regulated in serum of diabetic and in IOMe-AG538-injected rats (B).

The miRNA expression levels were measured in the blood serum of HFD/STZ-induced, IOMe-injected non-diabetic and IOMe-injected diabetic rats. The expression levels of miR-107, miR-181c, miR-103, miR-101, miR-29a, miR-21 and miR-9 were significantly down regulated in the blood serum of diabetic and IOMe-injected rats (Fig 5A) whereas, the expression levels of these miRNAs are normally high. The expression levels of miR-34a and miR-26b were significantly up regulated in serum of diabetic and IOMe-injected rats (Fig 5B), whereas the expression levels of these miRNAs are normally low.

## Discussion

In the present study, oxidative profile was investigated by using two parameters: typical oxidative stress parameters (MDA, NO and XO levels) and the antioxidant capacity parameters (SOD, GPx and GST enzyme activities and GSH level). The results demonstrated a high elevation in XO, NO levels, and TBARS level, which determined by a significant elevation in MDA content in the brain of STZ-induced diabetes compared to control brains. Also, the levels of enzymatic and non-enzymatic antioxidant parameters showed a significant decline in the brain of diabetic rats. The pressure from oxidative stress in combination with lowered antioxidant defense creates a harmful combination that disrupts functions and damage cells, which lead to loss of synapse and cell death. STZ promoted the generation of free radicals and lipid peroxidation in the brain *via* the XO pathway. These results run parallel with previous studies that linked between amyloid plaques formation and oxidative stress including lipid peroxidation markers such as MDA [38] and hyperglycemia activates multiple signaling pathways, which lead to increased ROS generation and induce insulin resistance [39]. Since oxidative stress started early in the disease progression, it is likely that treatment will be most effective if started early as well, before other damaging process takes over in “point of no return”. It is well confirmed in our results that the treatment of diabetic rats with NSO reversed the activities of the antioxidants effectively and reduced the oxidative stress significantly. Therefore, it is reasonable to assume that NSO treatment improves the oxidative balance of brain of T2DM because NSO was able to reduce the levels of XO, NO and MDA and free radical generation and increase levels of antioxidant parameters. The increase in SOD and GSH activities act as a compensatory mechanism for prolonged overproduction of oxidative stress and free radicals [40].

It was hypothesized that TNF-α educes antagonistic activity toward insulin due to its ability to augment IRS phosphorylation on Ser/Thr residues that leads to an insulin resistance [41]. The inhibition of insulin signaling pathway via TNF-α lead to suppression of the regulatory enzymes of fatty acids and glucose capture producing hyperglycemia [42]. In addition, IL-6 causes insulin resistance due to the defect in IRS phosphorylation leading to decrease in gluconeogenesis and increased glycogenolysis [43]. It was also reported that there is a close integration between pathogenesis of AD and neuro-inflammation involves in the glial cells activation in neurodegenerative diseases such as AD [44]. In our experimental results, the HFD/STZ rats demonstrated an increased in serum levels of pro-inflammatory markers TNF-α and IL-6 as well as increased concentrations of brain pro-inflammatory cytokines IL-1β, IL-6 and TNF-α protein and NF-κBp65 gene expression levels. These findings are consistent with previous data, which suggested that the treatment by STZ caused promoted neuro-inflammatory cytokines and altered redox status in STZ-treated rats leading to neurodegeneration [45].

Interestingly, the combination therapy of NSO and hypoglycemic drugs (MT and GI) significantly reduced the levels of pro-inflammatory markers and inhibited iNOS levels and NO formation. This combination is effectively thought to improve insulin action in the brain of diabetic rats and reduced the incidence of AD. One of the hallmarks of this mechanism is impaired cholinergic neurotransmission [46]. It was previously known that the metabolism of glucose in brain is associated with acetylcholine synthesis [47] and the reduced glucose levels in the brain may have a negative effect on cognitive function [48]. Also, the insulin resistance is known as a highly related factor associated with depletion of cognitive function in the brain. The impaired insulin signaling pathway is considered to cause improper glucose metabolism in the brain [18]. The treatment of diabetic rats with NSO alone exhibited a significant decline in the AChE activities that may help in restoring the normal level of acetylcholine. This is associated with normalized the brain glucose level, which in turn may help in preserving the cognitive function in brain and regulate the neuronal development. This finding is consistent with a recent study that revealed that *Nigella sativa* can prevent the damage of spatial memory after HFD/STZ-administration and reduced the AChE activity [49]. So, the greater the activity of AChE, the less effective in the cholinergic system.

In addition, NSO treatment of STZ-diabetic rats decreased significantly the level of Aβ-42 (7-fold), these results in agreement with what was reported before [50] that TQ has neuro-protection potential against Aβ-42 in rat hippocampal and cortical neurons and thus it might be considered as a promising candidate for AD treatment. It is contradictory to what has been suggested that MT alone increased the production of beta amyloid and enhanced the chances of AD [51]. It was found that the combination of NSO and MT displayed a significant decrease in brain Aβ-42 levels (4.5-fold) more than the value when MT was used alone [52]. Furthermore, it has been suggested that GI acts as a novel anti-inflammatory agent that could modify the progression of neurodegenerative diseases that it can reduce Aβ-42 production [53]. Other data showed GI attenuated Aβ production via suppressing BACE1 activity in cortical neurons [54]. Here, the treatment with GI alone decreased the level of Aβ-42 in the brain of STZ-induced rats (1.9-fold). Interestingly, one of our novel findings is the beneficial effect of combination therapy of NSO and GI in reducing the Aβ-42 produced in the brain of STZ-diabetic rats (4.5-fold). So, NSO-GI may serve as a promising drug for the treatment of AD associated with diabetes and brain insulin resistance.

Since NSO has the most beneficial effects on reducing blood glucose level, this hypoglycemic effect may be accompanied by the decrease of AGEs formation and RAGEs expression. It was found that MT decreased AGEs through reduction of hypoglycemia indirectly via insulin-dependent mechanism directly [55]. Moreover, another study showed that MT treatment decreased the oxidative stress and has a hypoglycemic effect, reduced AGEs and Aβ production and has remarkable role in down-regulation of RAGEs expression. The down-regulation of toxic AGEs levels and RAGEs expression by GI was observed in our present study and in agreement with the previous studies [56]. Accordingly, we suggest that GI has a potential benefit for neurodegeneration in diabetic rats. Importantly, the using of combination therapy of either GI or MT with NSO impaired AGEs formation and down-regulated the gene expression of RAGEs in diabetic rat’s brain of our experimental model.

In fact, it has been demonstrated that the decreased phosphorylation of similar insulin signaling molecules shown in AD or T2DM patients brains was more severe in the brains of the patients with both AD and T2DM [57]. The decreased in insulin signaling, including altered kinase activity and IRS expression, in AD gets worse with disease progression [58]. The model of HFD/STZ in the current study demonstrated that lowered levels of Tyr phosphorylation of IRS1-pTyr612 associated with decreased protein levels of AKT1-pSer473, and GSK-3β-pSer9 as well as increased GSK-3β levels. It was demonstrated the disruption in the brain insulin signaling pathway is responsible for the limited insulin function and impaired glucose transport in in the brain tissues upon STZ-induction, resulting in brain insulin resistance (diabetic brain/T3D) [59].

The treatment of T2DM-induced impairment of insulin signaling pathway with NSO decreased insulin resistance and repaired brain insulin signaling pathway. This is demonstrated by increment protein levels of Tyr phosphorylation of IRS1-pTyr612 and elevated levels of AKT1-pSer473, GSK-3β-pSer9 with decreased GSK-3β levels. Importantly, TQ readily crosses the blood-brain barrier due to its small size and facilitates its neuro-protection role in the diabetic brain [60]. The signal of the phosphorylated form of insulin signaling pathway proteins was greater in *Nigella sativa*-treated animals [61].

The neuroprotective effect of MT exhibited via variety of mechanisms such as reducing phosphorylation of Tau proteins through inhibition of the ubiquitin-dependent degradation of PP2A resulted in an elevation of PP2A level and dephosphorylation of Tau in AD [52]. In our study, we found that the role of anti-diabetic drug GI treatment alone and in combination with NSO caused elevation of GSK3β phosphorylation at Ser9 and normalized the PP2A activity thus inhibited the phosphorylation of Tau protein. Taken together, our observations suggest that NSO, MT and GI in the brain of diabetic rats have a prolonged effect on the PP2A, which is likely to overcome GSK3β counter-regulation of Tau phosphorylation.

In the present study, neurodegeneration-associated AD was detected in T2D rats. Therefore, we focused on the amyloidogenic-associated parameters and determined whether dysfunctions of these parameters are associated with AD progression in T2D rats. Some miRNAs are possibly related to AD pathology and has been consistently identified as dysregulated in AD. Interestingly, miR-9, which is responsible for neuronal differentiation, neurogenesis and development of brain, is highly expressed in hippocampus while it is down-regulated in AD brains. It has been reported that addition of Aβ42 peptides to primary neuronal cell cultures has been shown to down-regulate this small RNA [62]. In addition, miR-107 down-regulated in an early stage of AD, as it has been proven that it targets BACE1, thus regulating amyloid production as well as neurofibrillary tangles [63]. Furthermore, miR-29a expression is inversely correlated with BACE1 and increased the amyloid *in vitro* production in neuronal cellular models [64]. Moreover, miR-29 has target specific sites on BACE1 mRNA and its down-regulation increases AD progression [65]. In contrast, NF-κB signaling transcriptionally repressed miR-29a/b [66]. Also, HFD/STZ was shown to activate NF-κB expression levels in rats [67]. Our study demonstrated a significant down-regulation in miR-29a expression in brain and sera of induced rats with increased in BACE1 expression as well as Aβ levels in rat brains of T2DM.

## Conclusion

**Fig 6:**
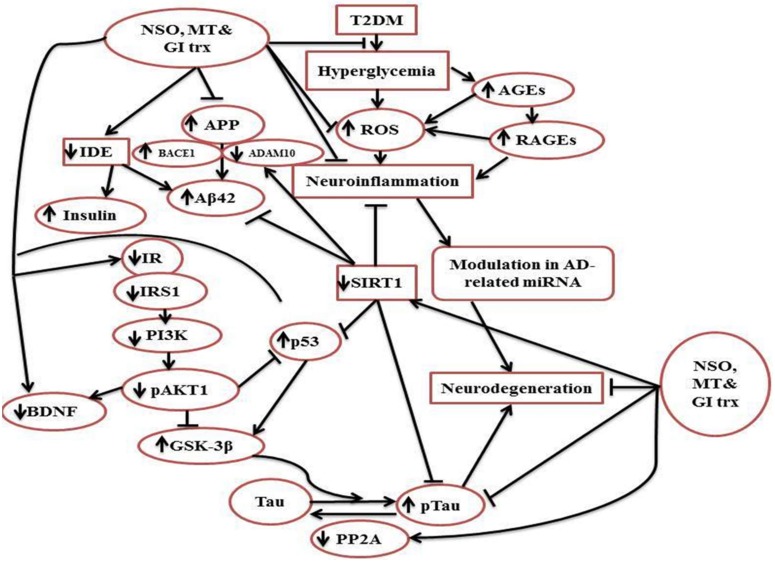
Schematic diagram for the effect of NSO combined with the anti-diabetic drugs MT and GI on brain insulin signaling in HFD/STZ-induced rats. This effect lowers the amelioration of ROS-induced T2DM and decreases the attenuation of the signaling molecules IR, IRS1, PI3K and AKT. This modifying effect results in Tau dephosphorylation, less neurodegeneration and modulation of AD-related miRNA.

The present study emphasized that insulin resistance of the brain of HFD/STZ-induced rats displays changes of AD-related biomarkers like APP, BACE1 expression and Aβ42 in addition to p-Tau protein levels with the elevation of inflammatory and oxidative stress markers. The observed abnormal expression of AD-associated miRNAs in the rat brains suggests that miRNAs participate in the process of AD and associated with T2DM. Hence, we suggest a promising use of miRNAs combined with other biomarkers in early diagnosis of AD for monitoring the progression of AD disease in T2DM-insulin resistance model. A schematic representation was designed (Fig 6) to summarize the modification of brain insulin signaling by NSO combined with the antidiabetic drugs.

## Supporting information

S1 File Manuscript data. (PDF)

S2 File Some diabetes parameters. (PDF)

## Abbreviations

AChE: acetylcholinesterase
AD: Alzheimer’s disease
ADAM10: A disintegrin and metalloprotease 10
AGE: advanced glycation end product
APP: amyloid precursor protein
Aβ: Amyloid beta
BACE1: Beta site AβPP cleaving enzyme
BDNF: brain derived neurotrophic factor
BW: body weight
DM: diabetes mellitus
DMSO: dimethyl sulfoxide
GI: glimepiride
GPx: glutathione peroxidase
GSH: glutathione
GSK-3β: glycogen synthase kinase 3 beta
GST: glutathione-s-transferase
HFD: high fat diet
IDE: Insulin degradation enzyme
IGF-1: insulin like growth factor-1
IL: interleukin
iNOS: inducible Nitric oxide synthases
IOMe: IOMe-AG538
IR: insulin receptor
IRS: insulin receptor substrate
MDA: malondialdehyde
miRNA: Micro RNA
MT: metformin
NF-κB: nuclear factor kappa-B
NO: nitric oxide
NSO: *Nigella sativa* oil
NSO-GI: combination of NSO and GI
NSO-MT: combination of NSO and MT
PGL: plasma glucose level
PI3K: phosphoinositide 3-kinase
PP2A: protein phosphatase 2A
RAGE: receptor of advanced glycation end product
ROS: reactive oxygen species
RT: reverse transcriptase
RT-PCR: real-time polymerase chain reaction
SIRT1: NAD-dependent deacetylase sirtuin-1
SOD: superoxide dismutase
STZ: streptozotocin
T2DM: type 2-diabetes mellitus
TBARS: thiobarbituric acid reactive substances
TNF-α: tumor necrosis factor-alpha
XO: xanthine oxidase
