# Supplementary material for: Oxidative stress and expression of insulin signaling proteins in the brain of diabetic rats: Role of Nigella sativa oil and antidiabetic drugs
Source: PLoS One. 2017 May 15;12(5):e0172429. doi: 10.1371/journal.pone.0172429 (PMC5432169; doi:10.1371/journal.pone.0172429)
Supplement: S1 File — (PDF) [file pone.0172429.s001.pdf]

S1 Table 1: Primer sequences and products size of target genes in expected PCR products for semi-quantitative RT-PCR.

Gene	Primer sequence	PCR program	Size (bp)
**APP**	F- 5′-AGAGGTCTACCCTGAACTGC-3′<br>R- 5′-ATCGCTTACAAACTCACCAAC-3′	94/30/55/30/72/60/35	154
**BACE1**	F: 5′-CGGGAGTGGTATTATGAAGTG-3′<br>R: 5′-AGGATGGTGATGCGGAAG-3′	94/30/60/30/72/60/30	320
**RAGE**	F- 5′-CTGGATGCTAGTCCTCAGTCTG-3′<br>R- 5′-CCTTTGCCATCAGGAATCAGAG-3′	94/60/58/60/72/60/30	500
**BDNF**	F- 5′-ATGGGACTCTGGAGAGCGTGAA-3′<br>R- 5′-CGC CAGCCA ATTCTC TTT TTGC-3′	94/30/60/30/72/60/30	574
**SIRT1**	F: 5′-CTTTGCCTCATCTGCATTTT-3′<br>R: 5′-ATTAGGCCAGCATTTTCTCA-3′	94/60/59/60/72/90/30	490
**ADAM10**	F-5′- GTTAATTCTGCTCCTCTCCTGG-3′<br>R- 5′-TGGATATCTGGGCAATCACAGC-3′	94/30/55/30/72/90/30	703
**NF-κB-p65**	F: 5'-GCGCATCCAGACCAACAATAA-3'<br>R: 5'-GCCGAAGCTGCATGGACACT-3'	95/30/56/30/72/30/35	424
**p53**	F: 5'-ATGGAGGAGTCACAGTCGGATA-3'<br>R: 5'-GACTTCTTGTAGATGGCCATGG-3'	94/60/52/60/72/60/30	500
**β-actin**	F- 5′-GCCATGTACGTAGCCATCCA-3′<br>R- 5′-GAACCGCTCATTGCCGATAG-3′	94/30/58/30/72/30/35	372

S1 Table 2: changes in oxidative stress parameters in diabetic rat brain after the course of treatment with NSO, antidiabetic drugs, combination therapy and I-OMeAG538. Values are expressed as mean± SE (n=8). Significance: $^{*}p<0.05$ (compared to control) and $^{\#}p<0.05$ (compared to diabetics).

Groups	TBARS (mmol/mg protein)	XO (µmol/hr/mg protein)	NO (µM/mg protein)
**Control**	302 ± 16.50$^{\#}$	17.20 ± 2.10$^{\#}$	553 ± 10.20$^{\#}$
**D**	1789 ± 31.80*	110.79 ± 3.60*	1254 ± 57.40*
**DMSO**	872 ± 8.25$^{*\#}$	63.95 ± 3.60$^{*\#}$	920 ± 30.90$^{*\#}$
**NSO**	424 ± 26.70$^{*\#}$	13.47 ± 0.68$^{\#}$	693 ± 28.80$^{*\#}$
**MT**	491 ± 33.60$^{*\#}$	30.30 ± 1.50$^{*\#}$	632 ± 31.60$^{*\#}$
**NSO-MT**	358 ± 14.50$^{\#}$	31.70 ± 1.45$^{*\#}$	666 ± 20.40$^{*\#}$
**GI**	504 ± 20.00$^{*\#}$	39.90 ± 3.80$^{*\#}$	839 ± 32.70$^{*\#}$
**NSO-GI**	331 ± 11.00$^{\#}$	23.16 ± 1.65$^{\#}$	628 ± 11.40$^{*\#}$
**D-NSO**	586 ± 27.40$^{*\#}$	36.60 ± 2.90$^{*\#}$	535 ± 17.20$^{\#}$
**D-MT**	554 ± 24.40$^{*\#}$	47.80 ± 5.80$^{*\#}$	690 ± 27.90$^{*\#}$
**D-GI**	650 ± 27.00$^{*\#}$	40.70 ± 3.57$^{*\#}$	592 ± 19.10$^{\#}$
**D-NSO-MT**	555 ± 26.80$^{*\#}$	38.25 ± 1.90$^{*\#}$	617 ± 11.50$^{*\#}$
**D-NSO-GI**	492 ± 33.00$^{*\#}$	47.80 ± 2.66$^{*\#}$	519 ± 7.70$^{\#}$
**IOMe**	1089 ± 20.70$^{*\#}$	82.50 ± 2.20$^{*\#}$	990 ± 18.70$^{*\#}$
**IOMe-NSO**	328 ± 23.30$^{\#}$	30.30 ± 2.08$^{*\#}$	578 ± 18.60$^{\#}$
**D-IOMe**	1060 ± 28.00$^{*\#}$	95.55 ± 5.00$^{*\#}$	927 ± 34.60$^{*\#}$
**D-IOMe-NSO**	528 ± 14.30$^{*\#}$	54.60 ± 1.25$^{*\#}$	690 ± 19.20$^{*\#}$

S1 Table 3: Antioxidant markers alterations in the brain of diabetic rats and after treatment with NSO, antidiabetic drugs, combination therapy and I-OMeAG538. Values are expressed as mean± SE (n=8). Significance: *p<0.05(compared to control) and $^{\#}$p<0.05 (compared to diabetics).

Groups	GSH (µg/mg protein)	GPx (nmol/min/mg protein)	GST (nmol/min/mg protein)	SOD (U/mg protein)
**Control**	3000 ± 20.67$^{\#}$	5670 ± 13.30$^{\#}$	1880 ± 14.00$^{\#}$	8610 ± 28.40$^{\#}$
**D**	1175 ± 14.10*	400 ± 6.55*	450 ± 11.00*	2460 ± 12.50*
**DMSO**	1245 ± 16.70*	1370 ± 13.00$^{*\#}$	610 ± 9.00*	3230 ± 9.89$^{*\#}$
**NSO**	3165 ± 15.98$^{\#}$	13800 ± 18.95$^{*\#}$	2240 ± 12.00$^{*\#}$	10700 ± 25.6$^{*\#}$
**MT**	2075 ± 14.90$^{*\#}$	6460 ± 17.60$^{\#}$	1200 ± 15.50$^{*\#}$	5590 ± 14.10$^{*\#}$
**NSO-MT**	1850 ± 14.62$^{*\#}$	12400 ± 16.60$^{*\#}$	1000 ± 8.00$^{*\#}$	5110 ± 16.30$^{*\#}$
**GI**	1812 ± 12.95$^{*\#}$	6960 ± 13.60$^{\#}$	1040 ± 6.00$^{*\#}$	5730 ± 21.60$^{*\#}$
**NSO-GI**	1900 ± 12.67$^{*\#}$	6720 ± 12.40$^{\#}$	1140 ± 6.20$^{*\#}$	6130 ± 16.50$^{*\#}$
**D-NSO**	3112 ± 18.10$^{\#}$	10910 ± 13.40$^{*\#}$	1980 ± 8.00$^{\#}$	9020 ± 22.20$^{\#}$
**D-MT**	2225 ± 17.25$^{*\#}$	3590 ± 11.80$^{*\#}$	1190 ± 5.00$^{*\#}$	6710 ± 9.20$^{*\#}$
**D-GI**	1537 ± 15.53$^{*\#}$	6480 ± 10.30$^{\#}$	1000 ± 7.00$^{*\#}$	4660 ± 15.50$^{*\#}$
**D-NSO-MT**	1687 ± 12.26$^{*\#}$	7660 ± 9.40$^{*\#}$	1097 ± 5.39$^{*\#}$	6920 ± 11.00$^{*\#}$
**D-NSO-GI**	1337 ± 14.60$^{*\#}$	6110 ± 12.90$^{\#}$	967 ± 3.40$^{*\#}$	4230 ± 12.30$^{*\#}$
**IOMe**	1437 ± 13.75$^{*\#}$	2160 ± 10.97$^{*\#}$	540 ± 7.00*	3460 ± 6.10$^{*\#}$
**IOMe-NSO**	2025 ± 15.26$^{*\#}$	6800 ± 16.10$^{\#}$	9140 ± 8.00$^{*\#}$	5750 ± 22.20$^{*\#}$
**D-IOMe**	1325 ± 13.65*	1500 ± 11.37$^{*\#}$	560 ± 6.00*	3010 ± 14.60*
**D-IOMe-NSO**	1725 ± 14.11$^{*\#}$	5320 ± 12.20$^{\#}$	958 ± 6.11$^{*\#}$	5490 ± 14.00$^{*\#}$

S1 Table 4: Serum TNF-α and IL-6 levels in diabetic rats after treatment with NSO, antidiabetic drugs, combination therapy and I-OMeAG538. Values are expressed as mean± SE (n=**8**). Significance: $^{*}p<0.05$ (compared to control) and $^{\#}p<0.05$ (compared to diabetics).

Group	TNF-α (pg/ml)	IL-6 (pg/ml)
**Control**	$16.60 \pm 0.24^{\#}$	$100.00 \pm 2.11^{\#}$
**D**	$134.00 \pm 2.40^{*}$	$610.00 \pm 3.00^{*}$
**DMSO**	$75.90 \pm 1.90^{*}$	$402.00 \pm 5.01^{*\#}$
**NSO**	$14.00 \pm 0.32^{\#}$	$85.50 \pm 3.10^{*\#}$
**MT**	$16.10 \pm 0.93^{\#}$	$100.10 \pm 3.02^{\#}$
**NSO-MT**	$15.70 \pm 0.47^{\#}$	$110.50 \pm 3.00^{\#}$
**GI**	$23.90 \pm 1.29^{*\#}$	$182.50 \pm 3.00^{*\#}$
**NSO-GI**	$19.00 \pm 0.27^{*\#}$	$162.30 \pm 1.90^{*\#}$
**D-NSO**	$17.01 \pm 0.52^{\#}$	$120.70 \pm 2.91^{\#}$
**D-MT**	$24.43 \pm 0.83^{*\#}$	$188.10 \pm 3.94^{*\#}$
**D-GI**	$30.70 \pm 1.22^{*\#}$	$196.50 \pm 3.01^{*\#}$
**D-NSO-MT**	$18.90 \pm 0.24^{*\#}$	$122.50 \pm 2.63^{\#}$
**D-NSO-GI**	$22.60 \pm 0.22^{*\#}$	$250.40 \pm 3.99^{*\#}$
**IOMe**	$100.50 \pm 2.49^{*\#}$	$450.50 \pm 3.55^{*\#}$
**IOMe-NSO**	$45.80 \pm 1.13^{*\#}$	$198.50 \pm 2.03^{*\#}$
**D-IOMe**	$127.90 \pm 2.84^{*}$	$650.30 \pm 5.67^{*}$
**D-IOMe-NSO**	$57.20 \pm 1.25^{*\#}$	$200.50 \pm 3.69^{*\#}$

S1 Table 5: Neuro-inflammation cytokines levels in the brain of diabetic rats after treatment with NSO, antidiabetic drugs, combination therapy and I-OMeAG538. Values are expressed as mean± SE (n=8). Significance: $^{*}p<0.05$ (compared to control) and $^{\#}p<0.05$ (compared to diabetics).

Groups	TNF-α (pg/mg protein)	IL-6 (pg/mg protein)	IL-1β (pg/mg protein)	iNOS (pg/mg protein)
**Control**	14.60 ± 0.22#	106.62 ± 2.14#	11.50 ± 0.25#	10.16 ± 0.09#
**D**	95.50 ± 1.40*	572.46 ± 3.76*	96.00 ± 0.95*	79.40 ± 0.67*
**DMSO**	40.90 ± 1.10*#	314.50 ± 5.04*#	41.80 ± 0.33*#	38.30 ± 0.33*#
**NSO**	14.20 ± 0.36#	92.50 ± 3.60#	10.30 ± 0.23#	9.00 ± 0.19#
**MT**	17.10 ± 0.23#	128.10 ± 3.62*#	16.40 ± 0.23*#	11.60 ± 0.19#
**NSO-MT**	18.70 ± 0.17#	140.50 ± 3.06*#	13.50 ± 0.20#	10.40 ± 0.17#
**GI**	17.90 ± 0.29#	152.50 ± 3.72*#	19.70 ± 0.44*#	12.20 ± 0.14#
**NSO-GI**	19.20 ± 0.27*#	152.30 ± 1.91*#	15.80 ± 0.30#	12.20 ± 0.14#
**D-NSO**	16.00 ± 0.32#	162.70 ± 3.71*#	17.90 ± 0.32*#	13.40 ± 0.13#
**D-MT**	23.40 ± 0.33*#	178.10 ± 3.64*#	23.22 ± 0.34*#	23.90 ± 0.67*#
**D-GI**	22.70 ± 0.22*#	190.50 ± 3.11*#	25.30 ± 0.25*#	17.60 ± 0.25*#
**D-NSO-MT**	18.30 ± 0.24#	222.50 ±3.63*#	21.00 ± 0.44*#	13.40 ± 0.42#
**D-NSO-GI**	24.70 ± 0.22*#	240.40 ± 3.03*#	22.60 ± 0.50*#	21.24 ± 0.48*#
**IOMe**	66.50 ± 0.49*#	440.50 ± 4.55*	72.00 ± 0.95*#	62.10 ± 0.39*
**IOMe-NSO**	22.80 ± 0.13*#	216.50 ± 2.43*#	24.40 ± 1.00*#	13.70 ± 0.40#
**D-IOMe**	75.90 ± 0.84*	480.30 ± 5.46*	85.00 ± 0.83*	50.00 ± 0.60*
**D-IOMe-NSO**	37.20 ± 0.25*#	228.50 ± 3.68*#	25.20 ± 0.66*#	14.70 ± 0.39*#

S1 Table 6: AChE activities and glucose levels in the brain of diabetic rats and after treatment with NSO, antidiabetic drugs, combination therapy and I-OMeAG538-injection. Values are expressed as mean± SE (n=8). Significance: $^{*}p<0.05$ (compared to control) and $^{\#}p<0.05$ (compared to diabetics).

Groups	AChE (µmol/min/mg protein)	Glucose (mg/mg protein)
**Control**	$93.60 \pm 1.34^{\#}$	$0.78 \pm 0.04^{\#}$
**D**	$306 \pm 9.60^{*}$	$0.13 \pm 0.02^{*}$
**DMSO**	$255 \pm 6.98^{*}$	$0.25 \pm 0.01^{*}$
**NSO**	$68.8 \pm 5.20^{*\#}$	$0.71 \pm 0.09^{\#}$
**MT**	$116 \pm 4.90^{*\#}$	$0.59 \pm 0.01^{*\#}$
**NSO-MT**	$166 \pm 5.10^{*\#}$	$0.74 \pm 0.04^{\#}$
**GI**	$110 \pm 2.90^{*\#}$	$0.44 \pm 0.02^{*\#}$
**NSO-GI**	$131 \pm 1.40^{*\#}$	$0.46 \pm 0.01^{*\#}$
**D-NSO**	$81 \pm 3.30^{*\#}$	$0.74 \pm 0.09^{\#}$
**D-MT**	$100 \pm 6.60^{\#}$	$0.45 \pm 0.07^{*\#}$
**D-GI**	$146 \pm 6.87^{*\#}$	$0.52 \pm 0.01^{*\#}$
**D-NSO-MT**	$122 \pm 4.90^{*\#}$	$0.50 \pm 0.09^{*\#}$
**D-NSO-GI**	$123 \pm 4.70^{*\#}$	$0.49 \pm 0.01^{*\#}$
**IOMe**	$227 \pm 4.30^{*}$	$0.29 \pm 0.05^{*}$
**IOMe-NSO**	$149 \pm 5.20^{*\#}$	$0.51 \pm 0.03^{*\#}$
**D-IOMe**	$265 \pm 6.70^{*}$	$0.19 \pm 0.06^{*}$
**D-IOMe-NSO**	$121 \pm 4.80^{*\#}$	$0.45 \pm 0.09^{*\#}$

S1 Table 7: Alteration in AGEs levels in brain and in serum of diabetic rats after treatment with NSO, antidiabetic drugs, combination therapy and I-OMeAG538. Values are expressed as mean± SE (n=8). Significance: $^{*}p<0.05$ (compared to control) and $^{\#}p<0.05$ (compared to diabetics).

Groups	Brain AGEs (U/mg protein)	Serum AGEs (U/ml)
**Control**	$0.10 \pm 0.01^{\#}$	$2.00 \pm 0.09^{\#}$
**D**	$1.32 \pm 0.07^{*}$	$25.20 \pm 0.19^{*}$
**DMSO**	$0.49 \pm 0.06^{*\#}$	$11.70 \pm 0.1^{*\#}$
**NSO**	$0.07 \pm 0.01^{\#}$	$1.30 \pm 0.06^{*\#}$
**MT**	$0.082 \pm 0.01^{\#}$	$2.20 \pm 0.04^{\#}$
**NSO-MT**	$0.068 \pm 0.02^{\#}$	$1.80 \pm 0.02^{\#}$
**GI**	$0.18 \pm 0.05^{\#}$	$2.10 \pm 0.05^{\#}$
**NSO-GI**	$0.11 \pm 0.03^{\#}$	$1.94 \pm 0.09^{\#}$
**D-NSO**	$0.094 \pm 0.01^{\#}$	$0.95 \pm 0.03^{*\#}$
**D-MT**	$1.68 \pm 0.04^{*\#}$	$3.00 \pm 0.04^{*\#}$
**D-GI**	$2.26 \pm 0.05^{*\#}$	$5.60 \pm 0.04^{*\#}$
**D-NSO-MT**	$0.08 \pm 0.01^{\#}$	$1.20 \pm 0.06^{*\#}$
**D-NSO-GI**	$0.13 \pm 0.03^{\#}$	$3.80 \pm 0.07^{*\#}$
**IOMe**	$0.79 \pm 0.04^{*\#}$	$19.20 \pm 0.13^{*}$
**IOMe-NSO**	$0.26 \pm 0.03^{*\#}$	$3.30 \pm 0.09^{*\#}$
**D-IOMe**	$1.46 \pm 0.06^{*}$	$28.70 \pm 0.17^{*}$
**D-IOMe-NSO**	$0.30 \pm 0.03^{*\#}$	$5.60 \pm 0.04^{*\#}$

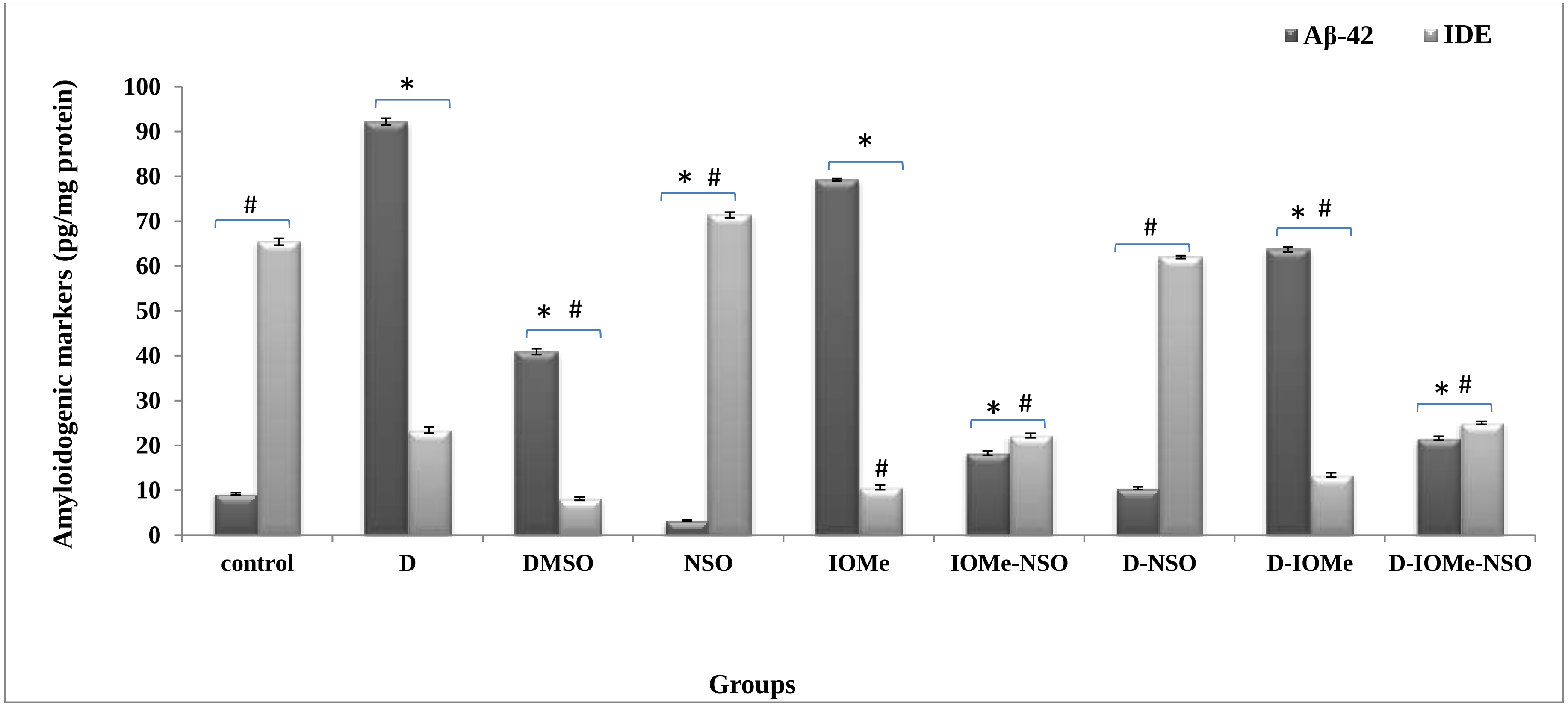
Aβ-42
IDE
Amyloidogenic markers (pg/mg protein)
100
90
80
70
60
50
40
30
20
10
0
#
*
* #
* #
*
#
* #
#
* #
* #
control
D
DMSO
NSO
IOMe
IOMe-NSO
D-NSO
D-IOMe
D-IOMe-NSO
Groups

S1 Fig. 1A

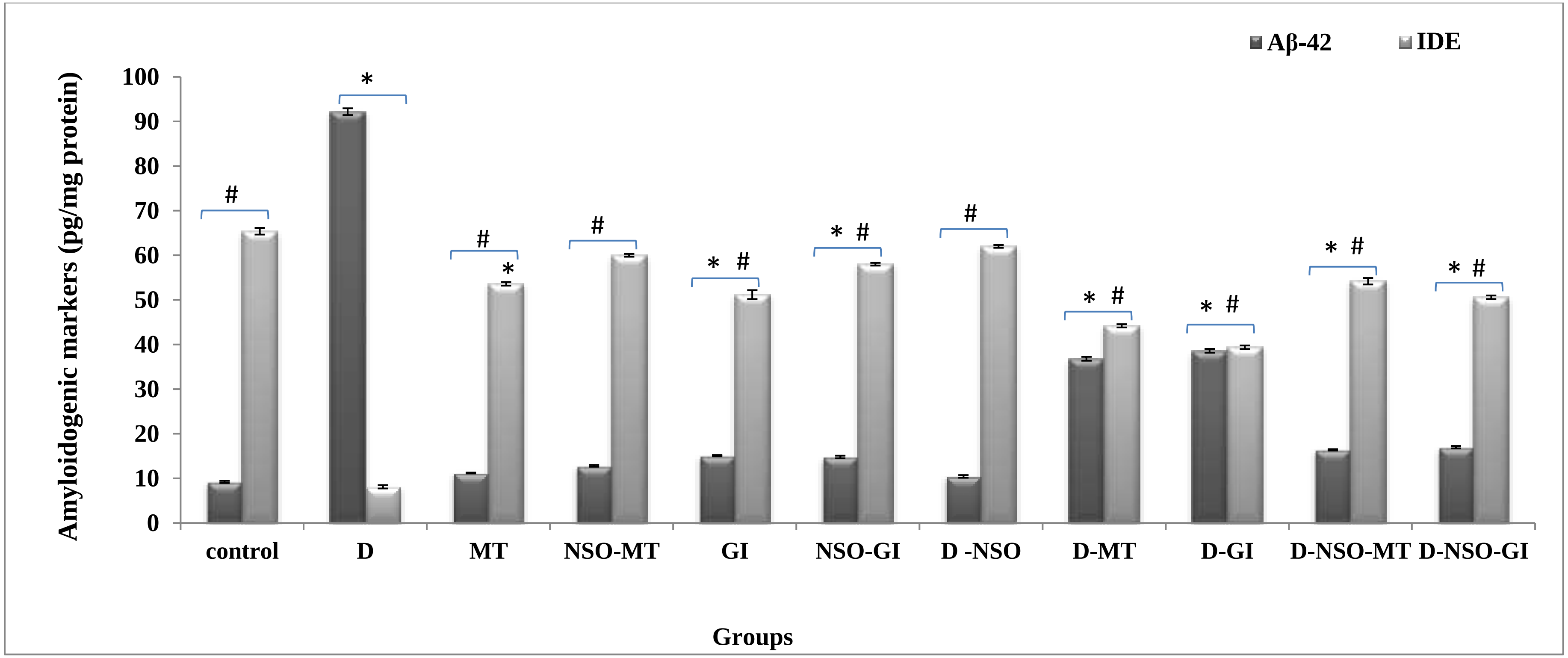

S1 Fig. 1B

S1 Fig. 1. Variations in brain Aβ-42 and IDE content. Aβ-42 & IDE levels in brain of diabetic and I-OMeAG-538-injected rats post NSO treatment (A). Aβ-42 & IDE levels in brain homogenate of diabetic rats after the course of treatment with NSO, reference drugs and combination therapy (B).

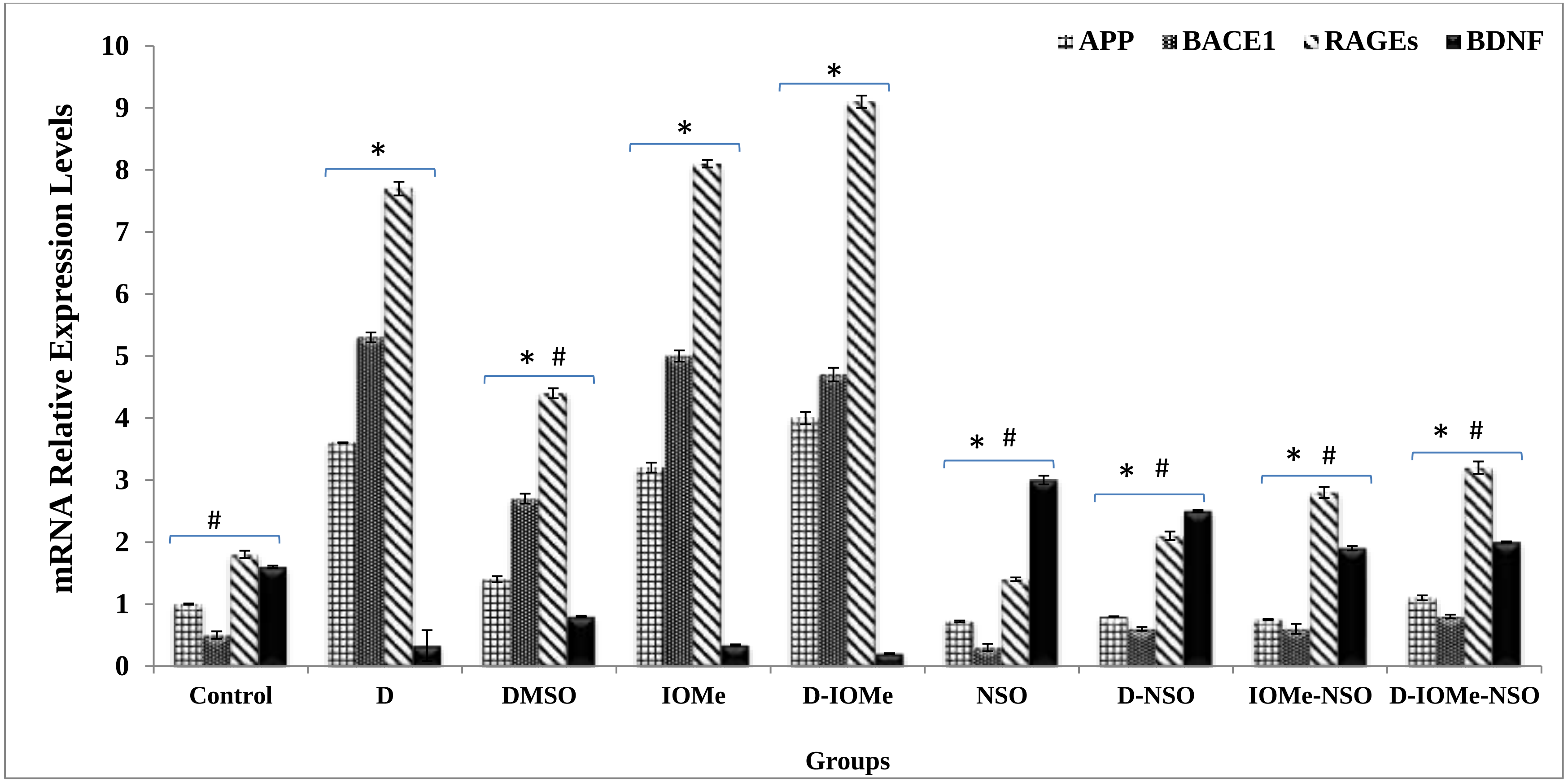
APP
BACE1
RAGEs
BDNF
mRNA Relative Expression Levels
10
9
8
7
6
5
4
3
2
1
0
#
*
* #
Control
D
DMSO
IOMe
D-IOMe
NSO
D-NSO
IOMe-NSO
D-IOMe-NSO
Groups

S1 Fig. 2A

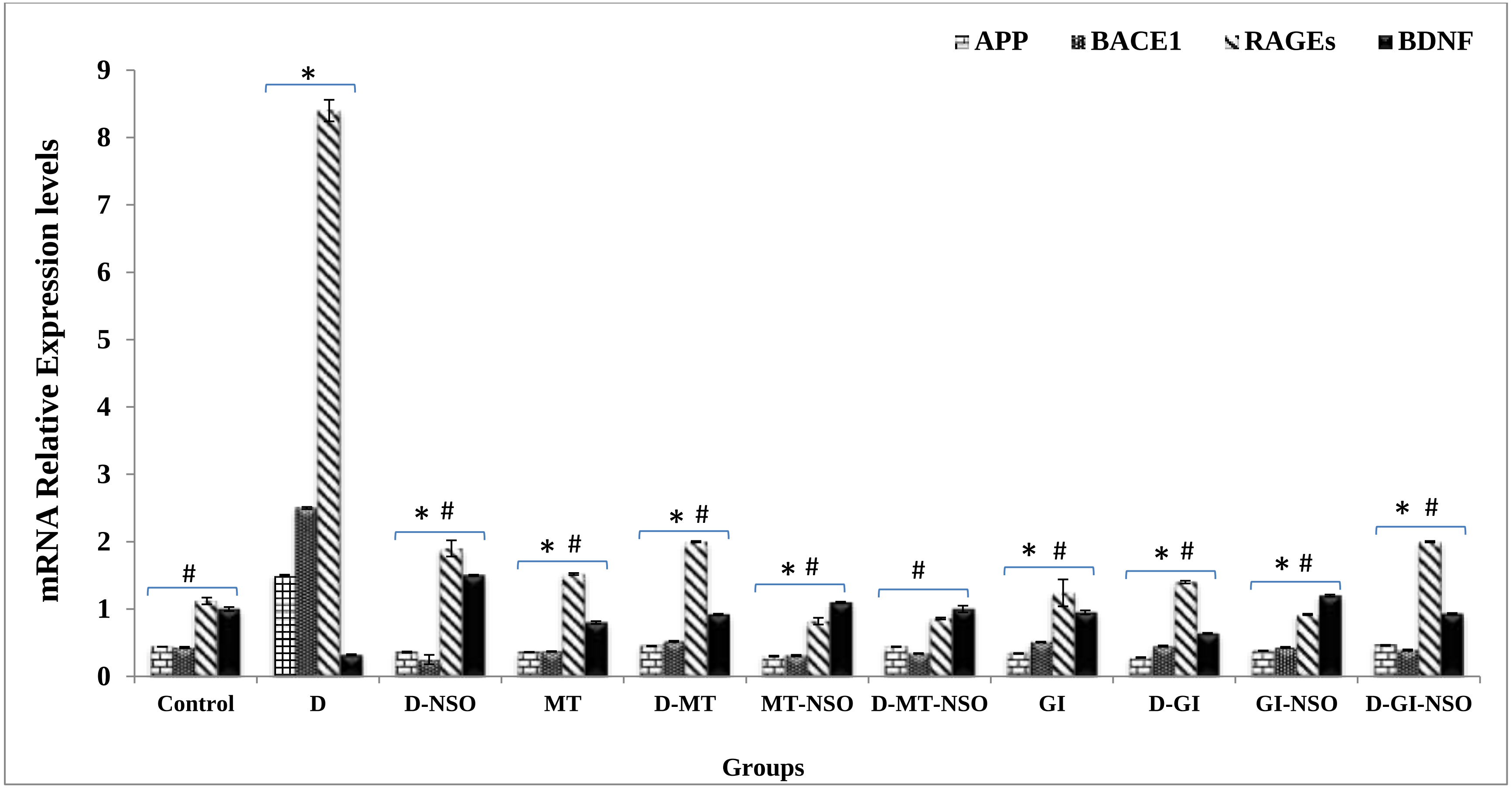
APP
BACE1
RAGEs
BDNF
mRNA Relative Expression levels
0
1
2
3
4
5
6
7
8
9
Control
D
D-NSO
MT
D-MT
MT-NSO
D-MT-NSO
GI
D-GI
GI-NSO
D-GI-NSO
Groups

S1 Fig. 2B

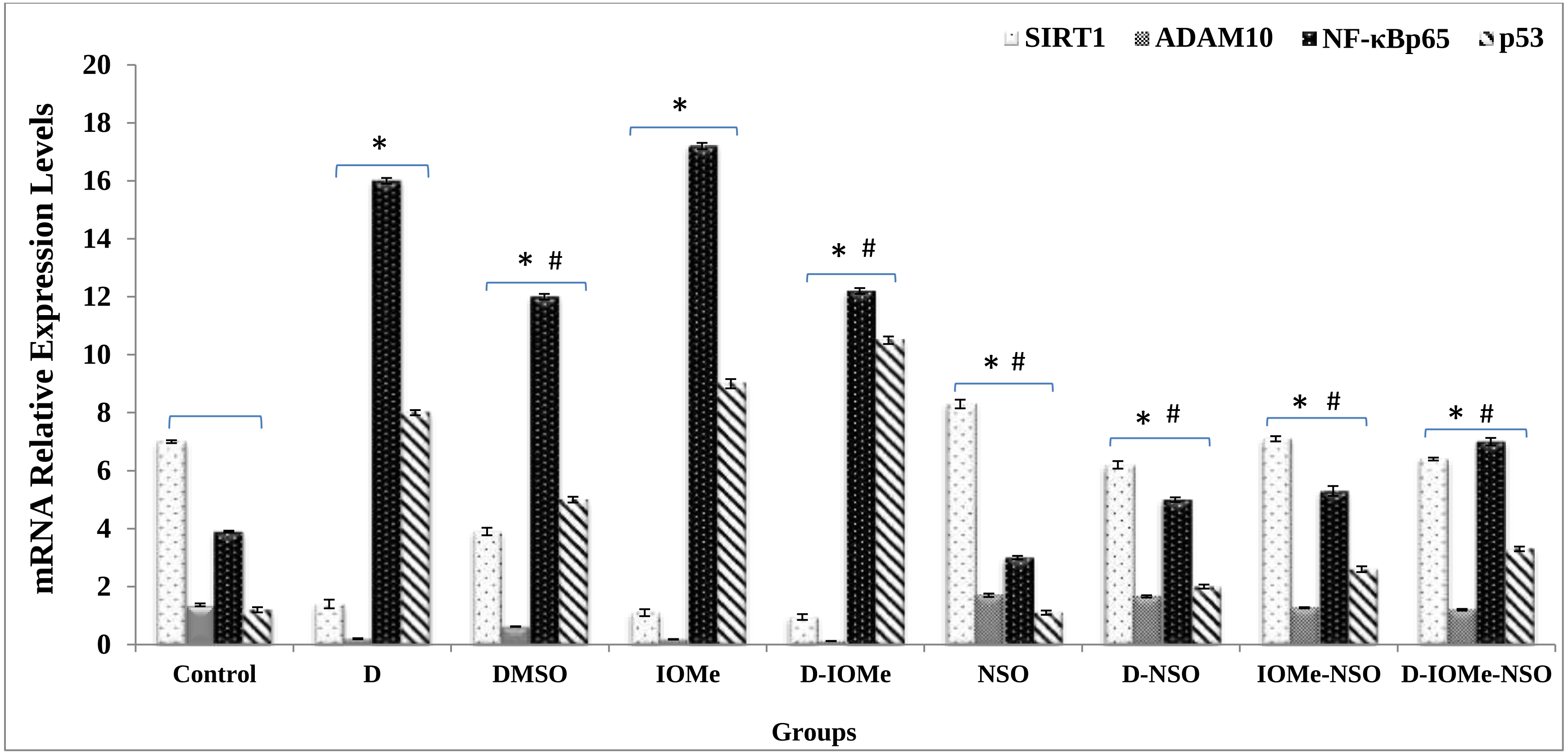
SIRT1
ADAM10
NF-κBp65
p53
mRNA Relative Expression Levels
20
18
16
14
12
10
8
6
4
2
0
*
* #
*
* #
* #
* #
* #
* #
Control
D
DMSO
IOMe
D-IOMe
NSO
D-NSO
IOMe-NSO
D-IOMe-NSO
Groups

S1 Fig. 2C

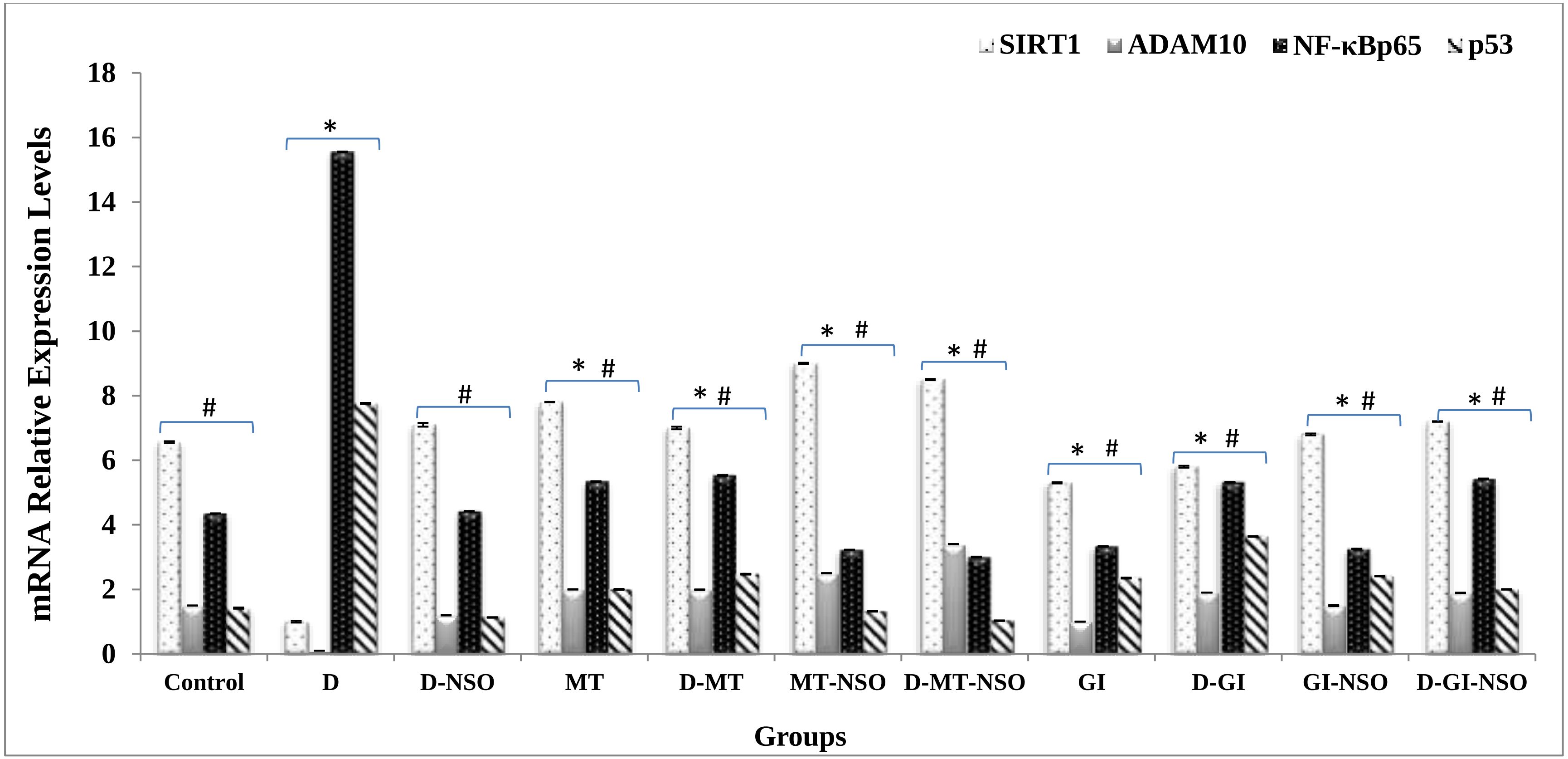
SIRT1
ADAM10
NF-κBp65
p53
mRNA Relative Expression Levels
0
2
4
6
8
10
12
14
16
18
Control
D
D-NSO
MT
D-MT
MT-NSO
D-MT-NSO
GI
D-GI
GI-NSO
D-GI-NSO
Groups

S1 Fig. 2D

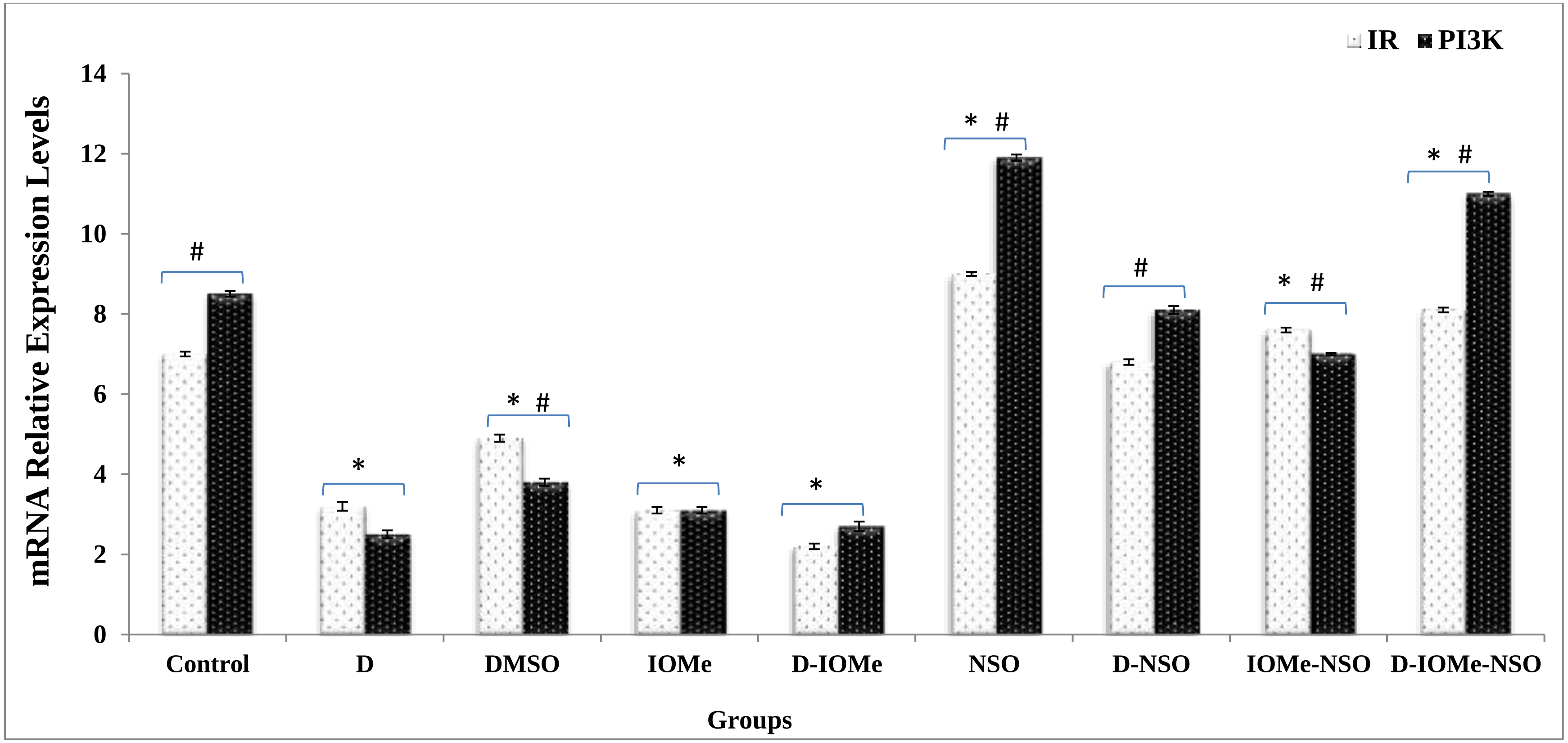
IR
PI3K
mRNA Relative Expression Levels
14
12
10
8
6
4
2
0
#
*
* #
*
*
* #
#
* #
* #
Control
D
DMSO
IOMe
D-IOMe
NSO
D-NSO
IOMe-NSO
D-IOMe-NSO
Groups

S1 Fig. 2E

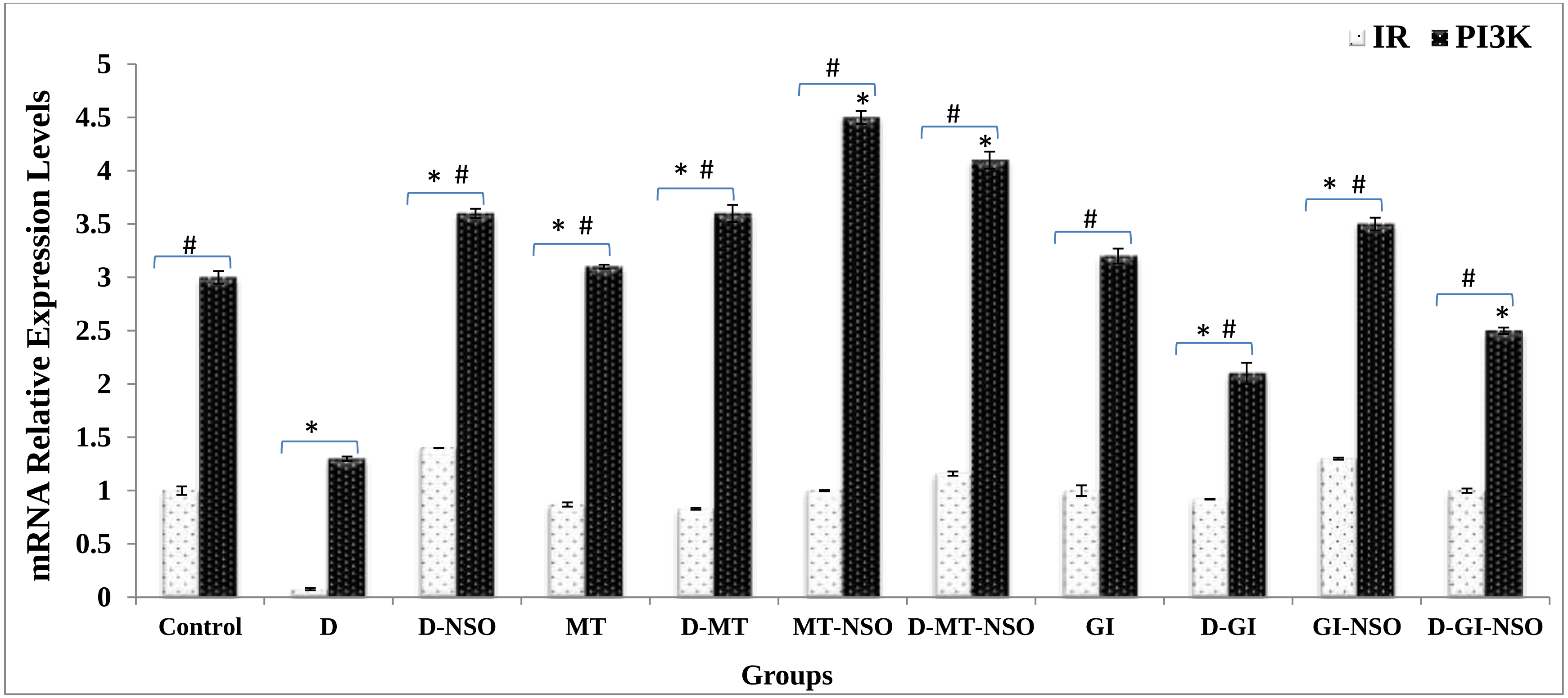

S1 Fig. 2F

S1 Fig. 2. Gene expression profiles of brain amyloidogenic, neuro-protection and insulin signaling biomarkers. mRNA levels by RT-PCR analysis for APP, BACE1, BDNF and RAGEs in brain tissues of diabetic and I-OMeAG538- injected rats versus NSO- treated rats (A). mRNA levels of APP, BACE1, BDNF and RAGEs in the brain of diabetic rats post the treatment with NSO, reference drugs and combination therapy (B). mRNA levels SIRT1, ADAM10, NF-κBp65 and p53 in the brain of diabetic rats, I-OMe-injected rats versus NSO treated rats (C). mRNA levels of SIRT1, ADAM10, NF-κBp65 and p53 in brain tissues of diabetic rats, and after treatment with NSO, reference drugs and combination therapy for (D). mRNA levels of IR and PI3K in the brain of diabetic rats, I-OMeAG538-injected rats versus NSO treated rats (E). mRNA levels of IR and PI3K in the brain of diabetic rats, and after treatment with NSO, MT, GI and combination therapy for IR and PI3K (F). β-actin was used as an internal control and quantification of bands using UVIBAND Image quantification software.

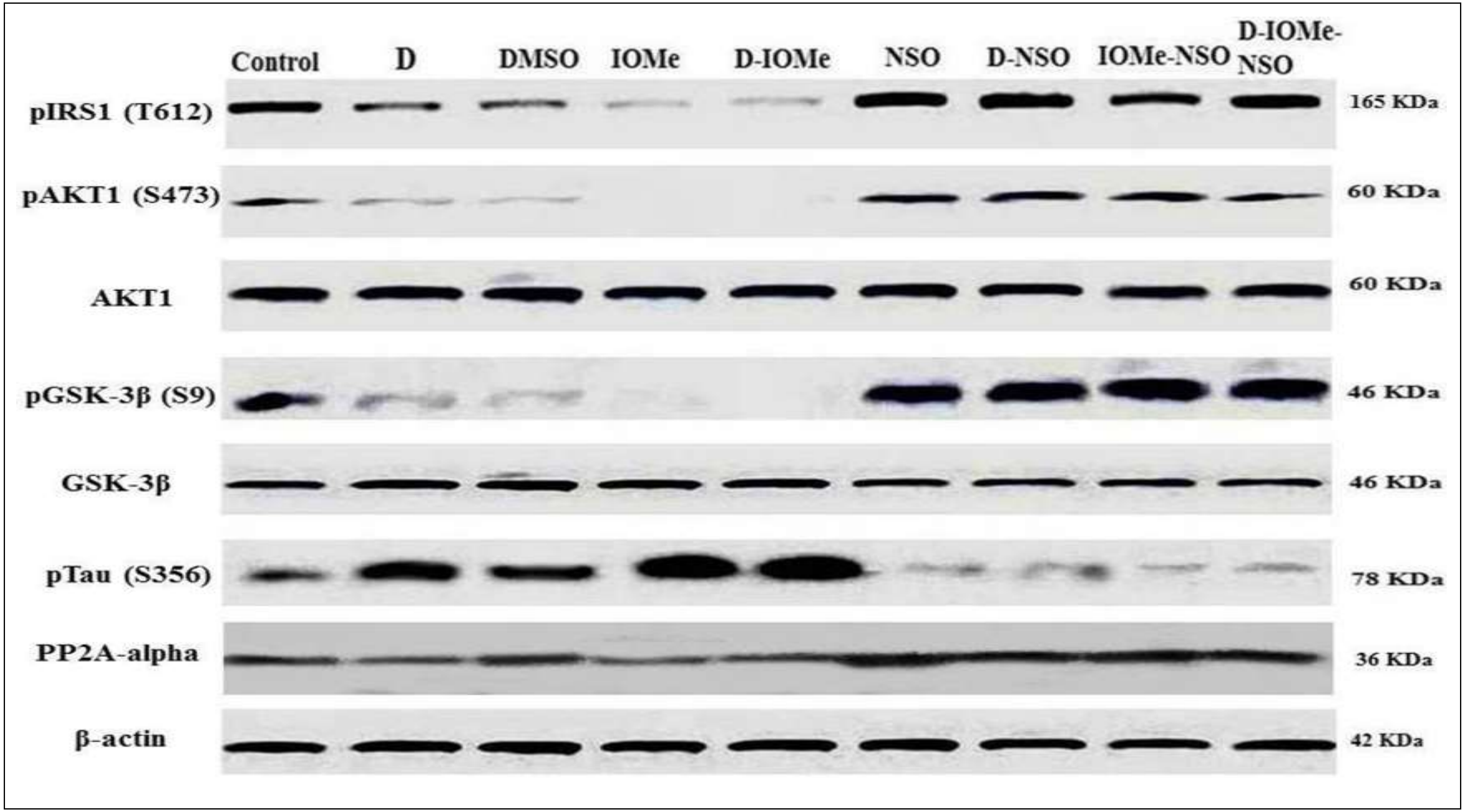
Control
D
DMSO
IOMe
D-IOMe
NSO
D-NSO
IOMe-NSO
D-IOMe-NSO
pIRS1 (T612)
pAKT1 (S473)
AKT1
pGSK-3β (S9)
GSK-3β
pTau (S356)
PP2A-alpha
β-actin
165 KDa
60 KDa
60 KDa
46 KDa
46 KDa
78 KDa
36 KDa
42 KDa

S1 Fig. 3A

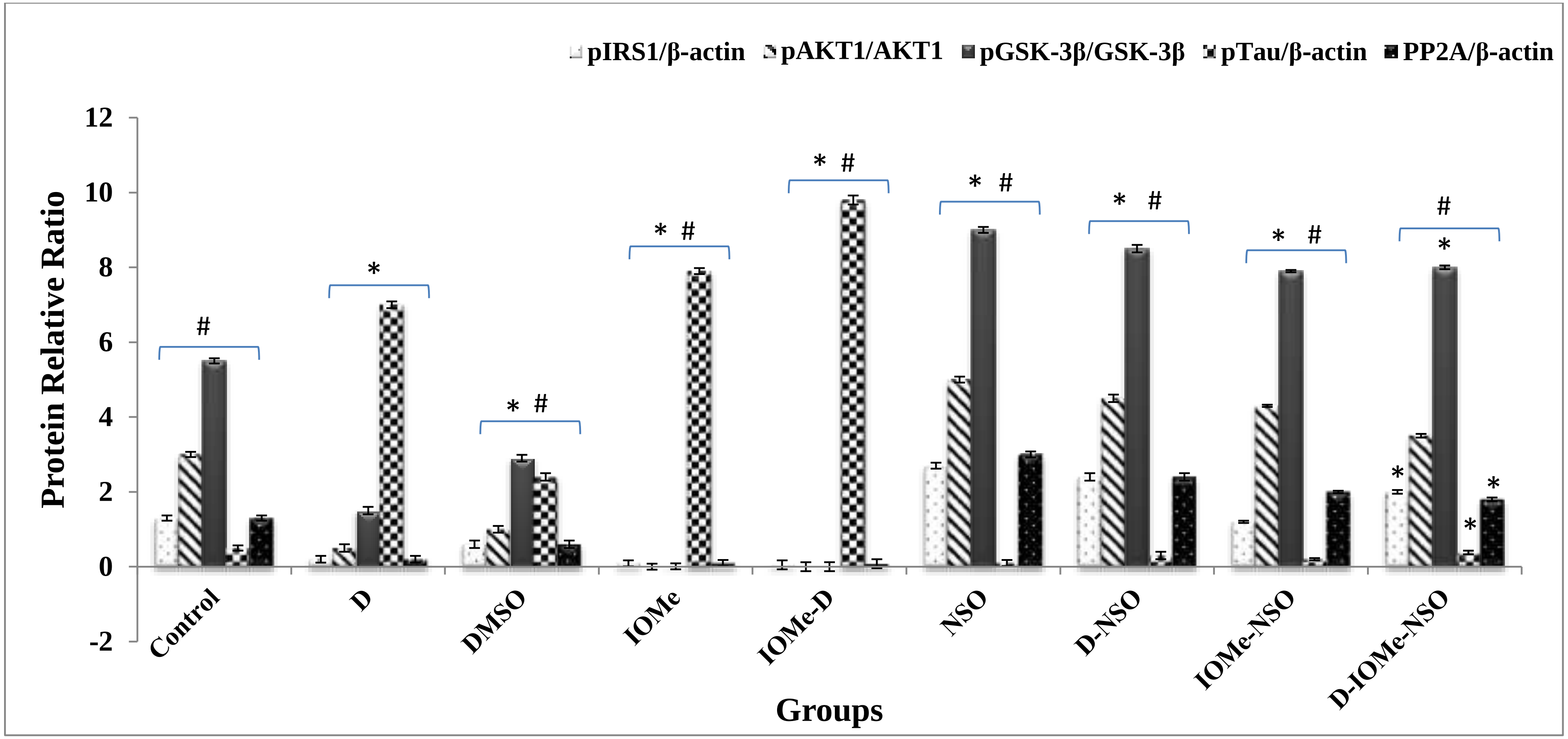
pIRS1/β-actin
pAKT1/AKT1
pGSK-3β/GSK-3β
pTau/β-actin
PP2A/β-actin
Protein Relative Ratio
12
10
8
6
4
2
0
-2
Control
D
DMSO
IOMe
IOMe-D
NSO
D-NSO
IOMe-NSO
D-IOMe-NSO
Groups

S1 Fig. 3B

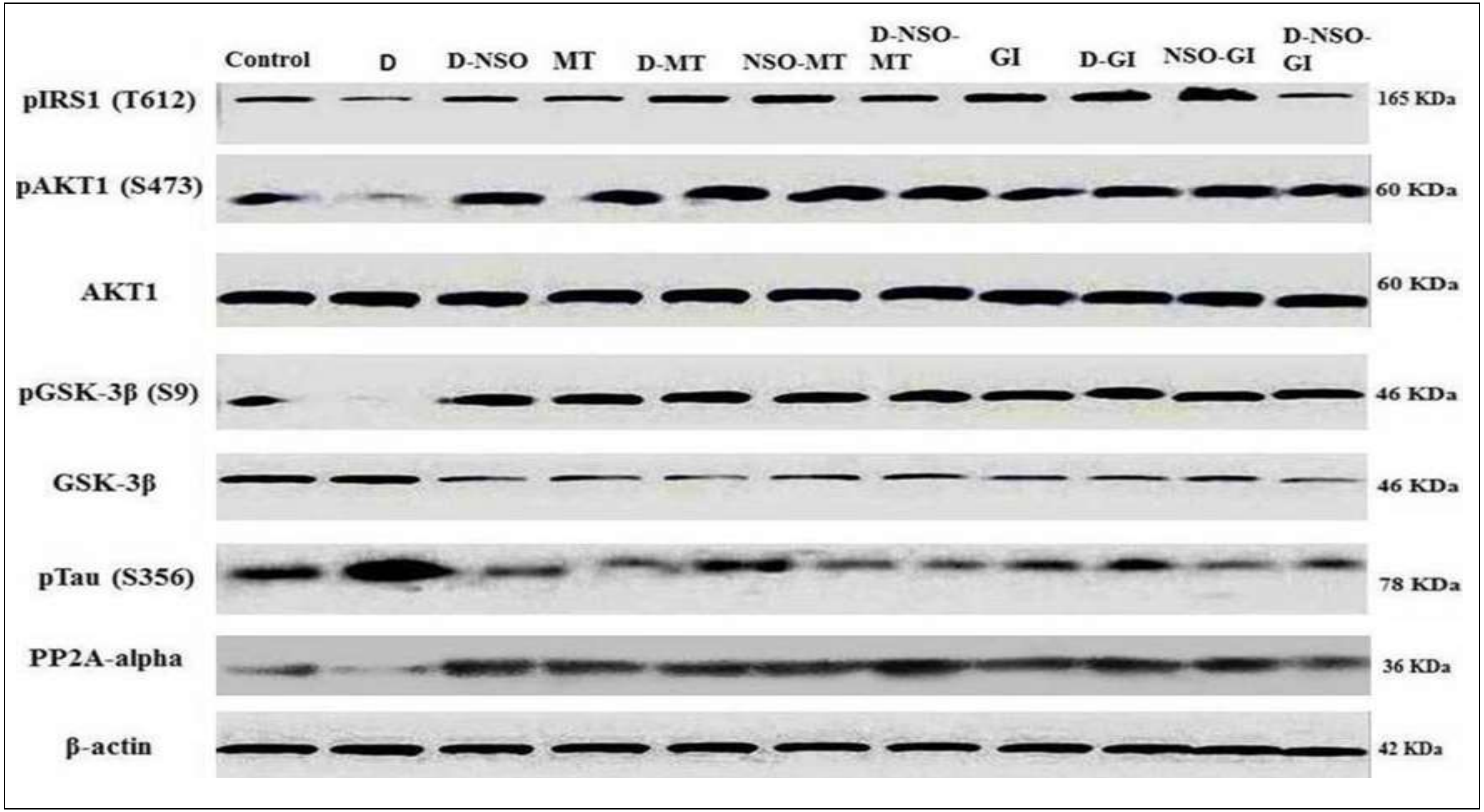
Control
D
D-NSO
MT
D-MT
NSO-MT
D-NSO-MT
GI
D-GI
NSO-GI
D-NSO-GI
pIRS1 (T612)
pAKT1 (S473)
AKT1
pGSK-3β (S9)
GSK-3β
pTau (S356)
PP2A-alpha
β-actin
165 KDa
60 KDa
60 KDa
46 KDa
46 KDa
78 KDa
36 KDa
42 KDa

S1 Fig. 3C

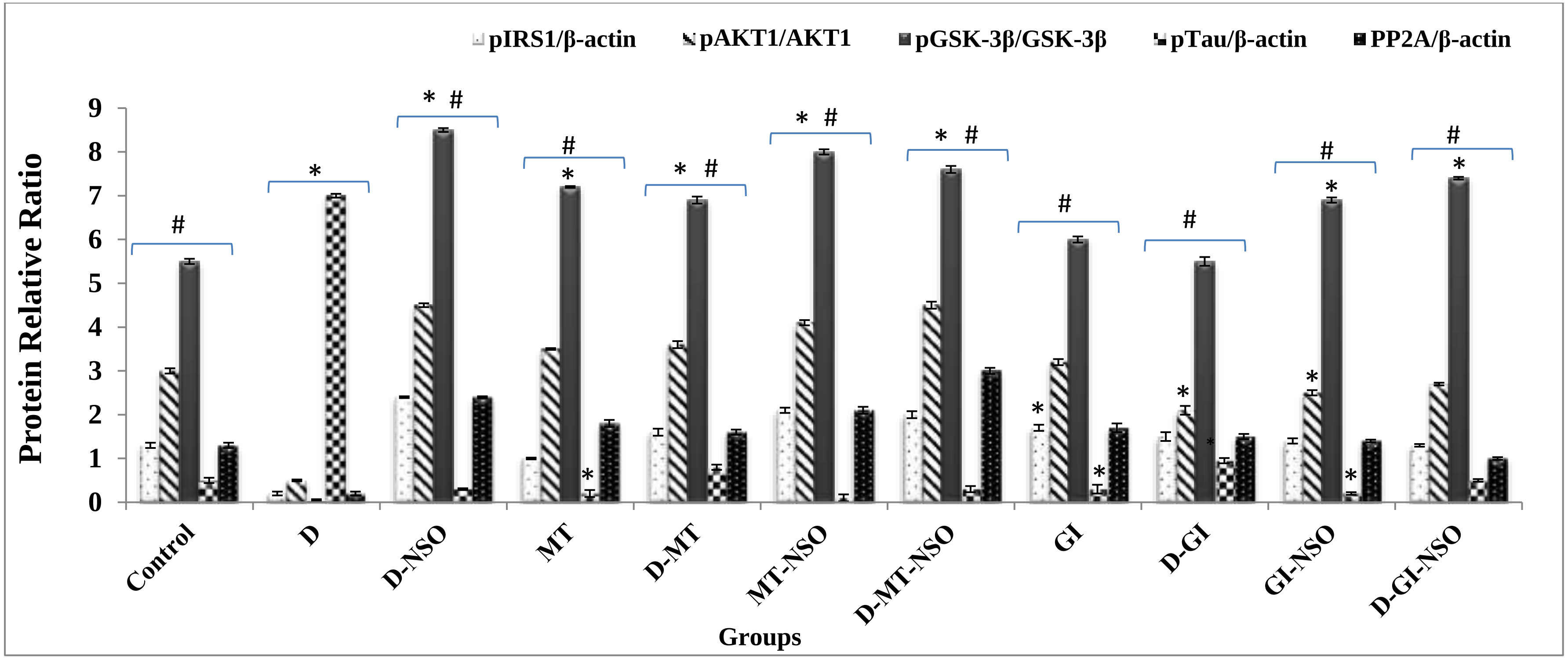

S1 Fig. 3D

S1 Fig. 3. Representative western blotting analyses of brain insulin-induced signaling molecules. Protein level profile of p-IRS1-Tyr612 was normalized to the total amount of β-actin protein and p-AKT1-Ser473 level was normalized to the total amount of AKT1. p-GSK3β-Ser9 was normalized to the total amount of GSK3β. p-Tau-Ser356 and PP2A-alpha levels were normalized to the total amount of β-actin protein in the brain tissue of diabetic rats injected with IOMe and treated with NSO (A). Protein profile of diabetic rats after the treatment with NSO, anti-diabetic drugs and combination therapy is shown as (C). The quantification of protein bands is represented as mean ± SE (n=5/group) (B) and (D).

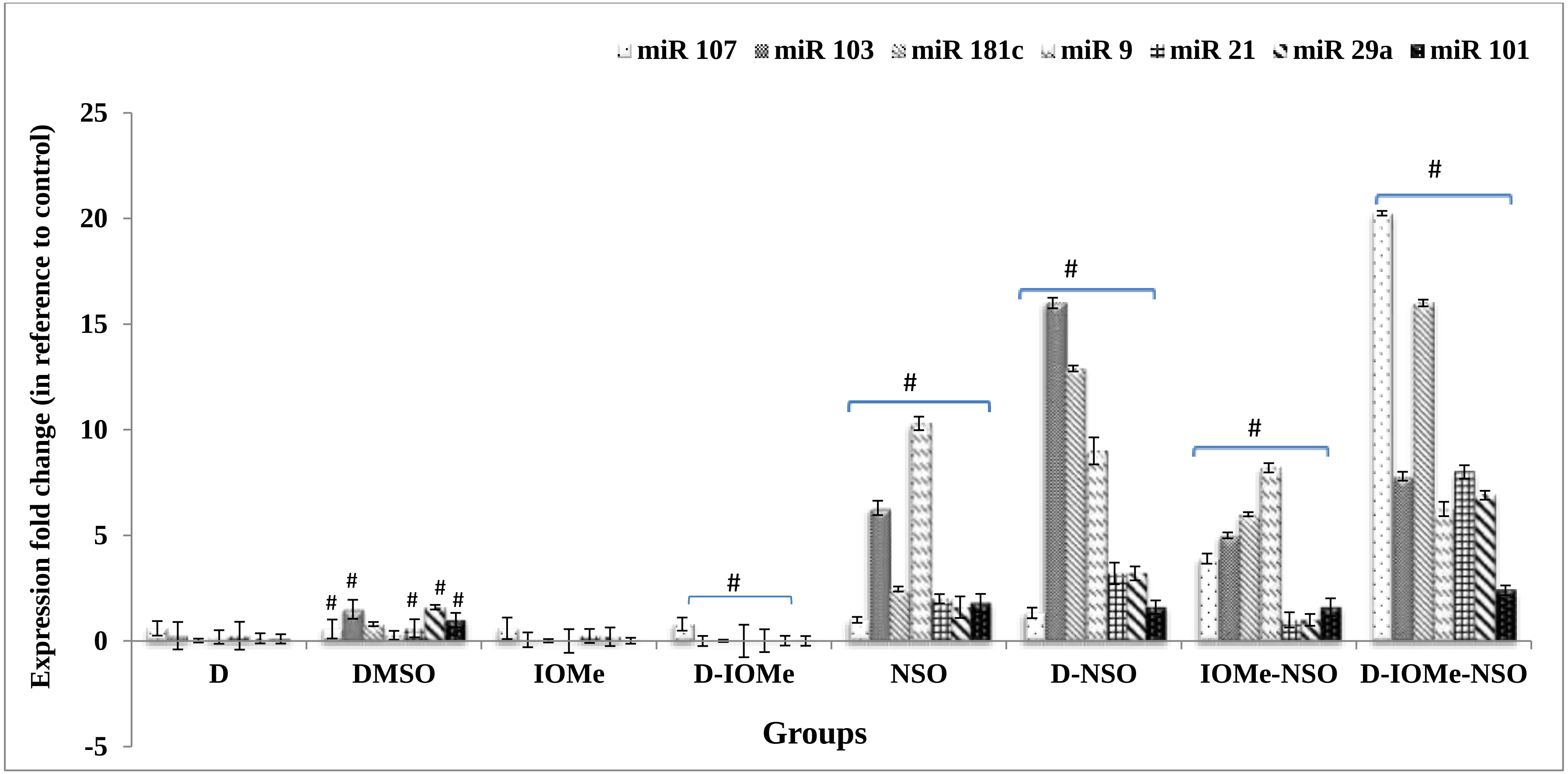
miR 107
miR 103
miR 181c
miR 9
miR 21
miR 29a
miR 101
Expression fold change (in reference to control)
25
20
15
10
5
0
-5
#
D
DMSO
IOMe
D-IOMe
NSO
D-NSO
IOMe-NSO
D-IOMe-NSO
Groups

S1 Fig. 4A

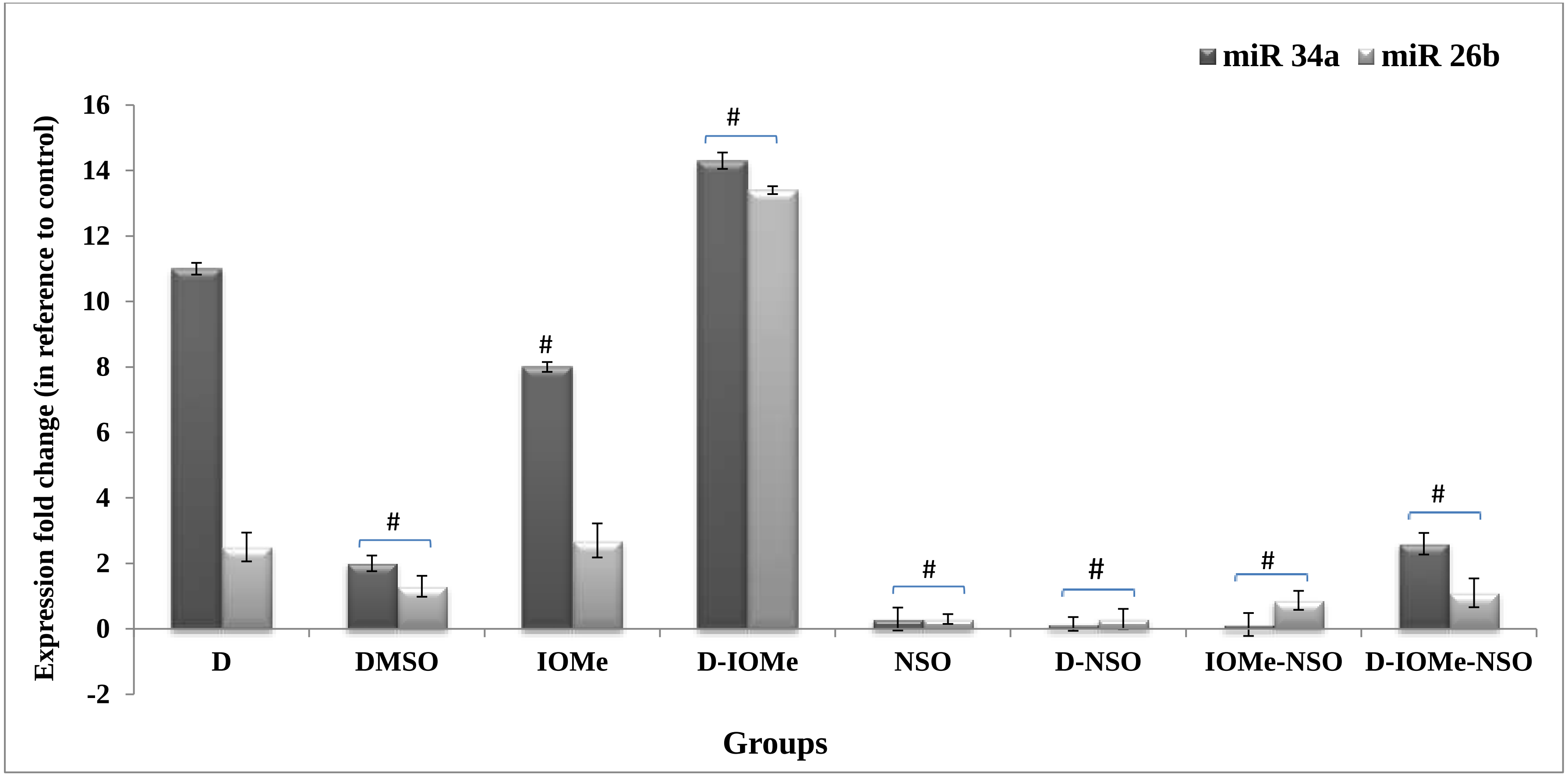

miR 34a
miR 26b
16
14
12
10
8
6
4
2
0
-2
Expression fold change (in reference to control)
#
#
#
#
#
#
#
D
DMSO
IOMe
D-IOMe
NSO
D-NSO
IOMe-NSO
D-IOMe-NSO
Groups

S1 Fig. 4B

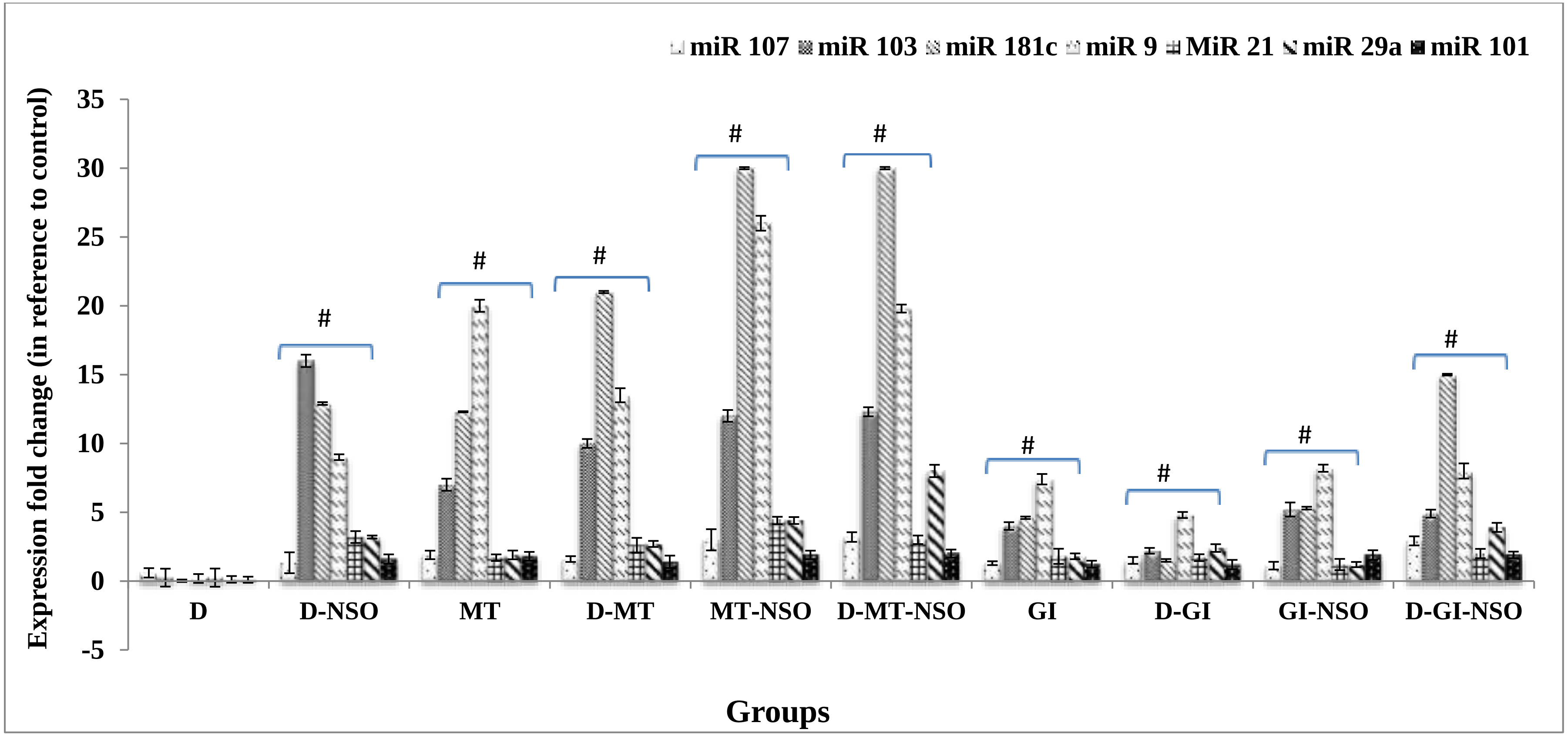

miR 107
miR 103
miR 181c
miR 9
MiR 21
miR 29a
miR 101
Expression fold change (in reference to control)
35
30
25
20
15
10
5
0
-5
#
D
D-NSO
MT
D-MT
MT-NSO
D-MT-NSO
GI
D-GI
GI-NSO
D-GI-NSO
Groups

S1 Fig. 4C

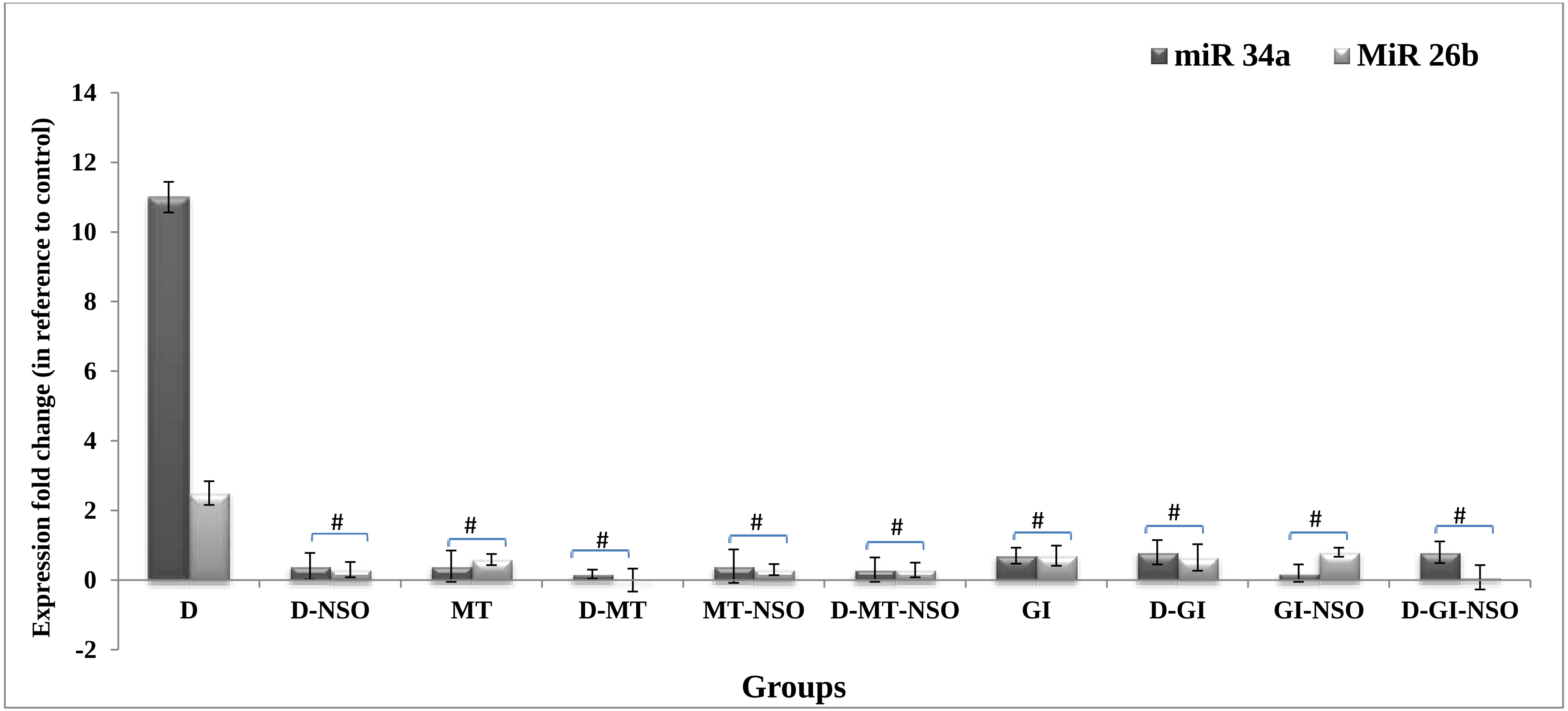

S1 Fig. 4D

S1 Fig. 4. Quantitative RT-PCR analyses of brain AD-related miRNAs expression profile. Alterations in the expression levels of AD-related miRNAs (107, 103, 9, 181c, 21, 101 and 29a) in the brain homogenate of experimental diabetic rats injected with IOMe and treated with NSO (A). Alterations in the expression level of miRNAs 34a and 26b in the brain of IOMe-injected diabetic rats and treated with NSO (B). Alterations in the expression levels of AD-related miRNAs (107, 103, 9, 181c, 21, 101 and 29a) in the brain of diabetic rats after treatment with NSO, anti-diabetic drugs and combination therapy (C). Alterations in the expression level of miRNAs 34a and 26b in the brain of experimental diabetic rats after treatment with NSO, anti-diabetic drugs and combination therapy (D). Significance is shown as (#p < 0.05) compared to diabetes-induced rats.

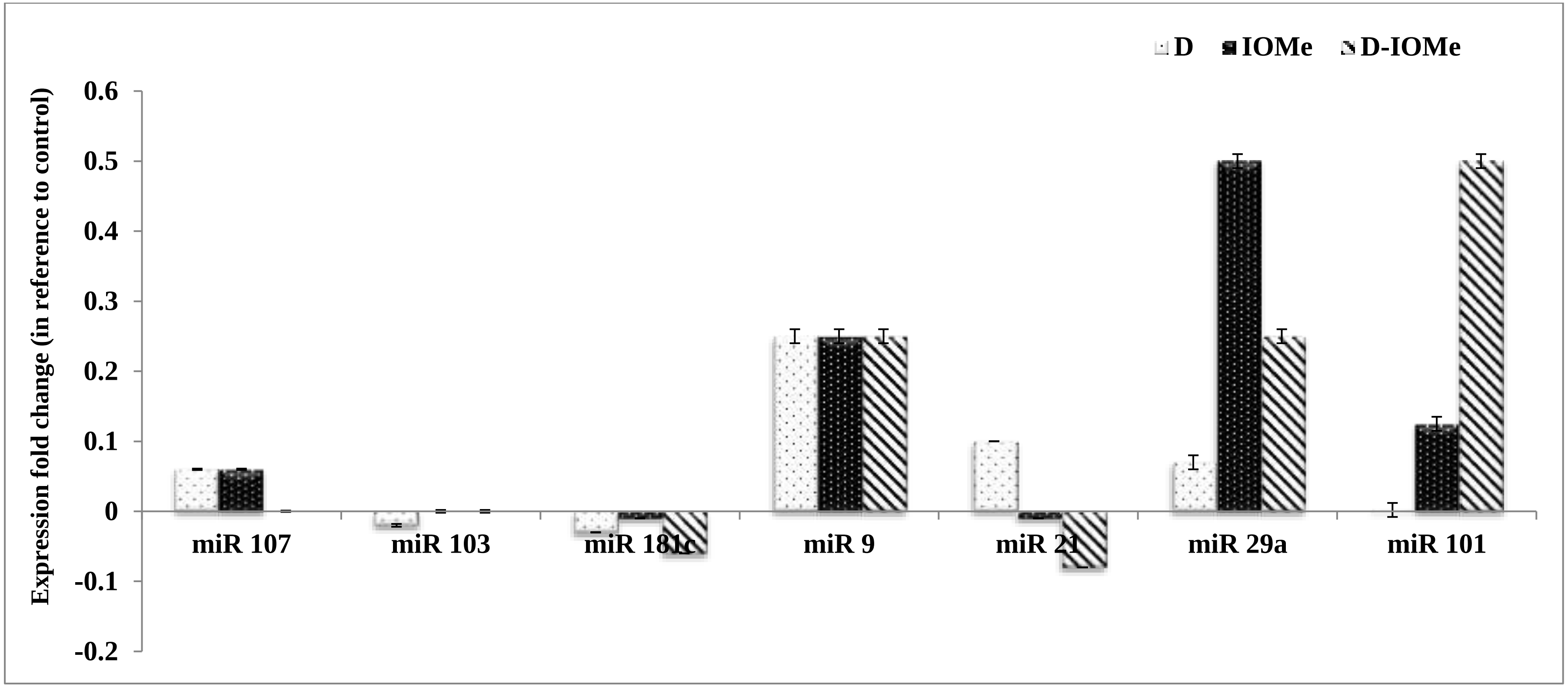
D
IOMe
D-IOMe
Expression fold change (in reference to control)
0.6
0.5
0.4
0.3
0.2
0.1
0
-0.1
-0.2
miR 107
miR 103
miR 181c
miR 9
miR 21
miR 29a
miR 101

S1 Fig. 5A

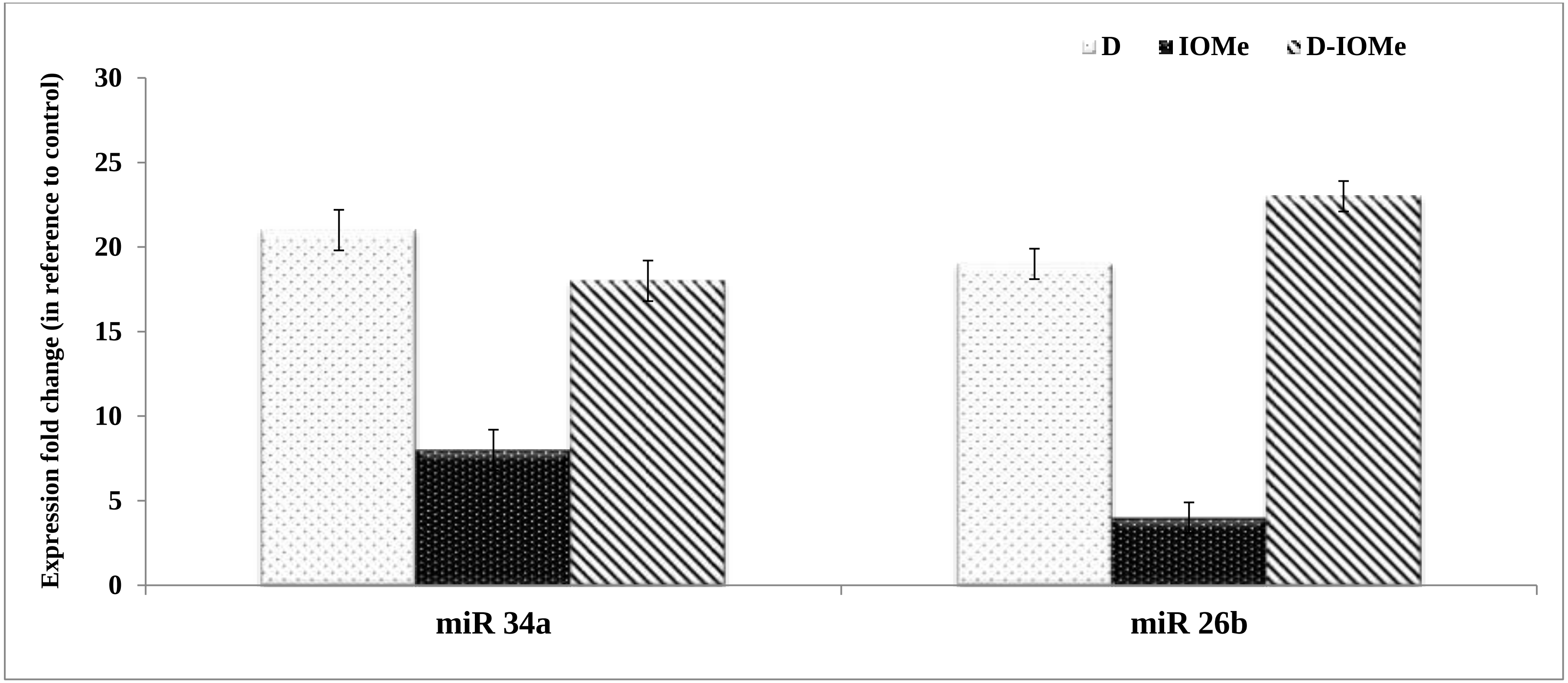

S1 Fig. 5B

S1 Fig. 5. Decreased miRNA expression levels in the blood serum of HFD/STZ-induced rats. The expression levels of miRNAs miR-107, miR-181c, miR-103, miR-101, miR-29a, miR-21 and miR-9 expression levels were down-regulated in the serum of diabetic rats and IOMe-injected rats (A). The expression levels of miRNAs miR-34a and miR-26b were up-regulated in serum of diabetic and in IOMe-AG538-injected rats (B).

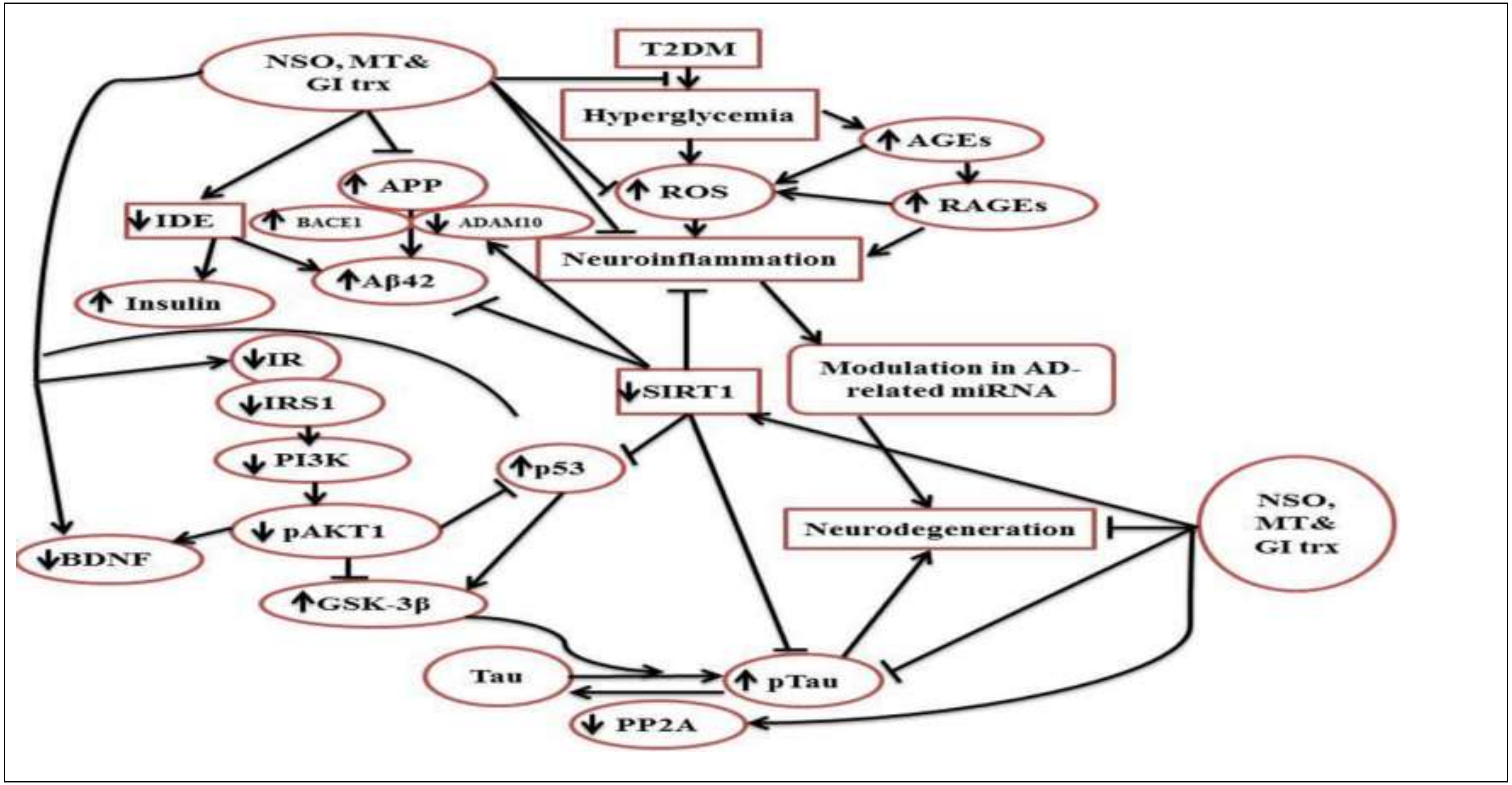

S1 Fig. 6. Schematic diagram for the effect of NSO combined with the anti-diabetic drugs MT and GI on brain insulin signaling in HFD/STZ-induced rats. This effect lowers the amelioration of ROS-induced T2DM and decreases the attenuation of the signaling molecules IR, IRS1, PI3K and AKT. This modifying effect results in Tau dephosphorylation, less neurodegeneration and modulation of AD-related miRNA.
