# Supplementary material for: Oxidative stress and expression of insulin signaling proteins in the brain of diabetic rats: Role of Nigella sativa oil and antidiabetic drugs
Source: PLoS One. 2017 May 15;12(5):e0172429. doi: 10.1371/journal.pone.0172429 (PMC5432169; doi:10.1371/journal.pone.0172429)
Supplement: S2 File — (PDF) [file pone.0172429.s002.pdf]

S2 Table 1. BGL, AI, AAI, Insulin, HOMA-IR and HOMA-β values after 21 days-treatment with NSO, reference drugs and I-OMeAG 538. Values are expressed as mean± SE (n=10). Significance: *p<0.05, **p<0.01, ***p<0.001 compared to control (data published by Balbaa *et al*., 2016 (Oxid Med Cell Longev. 2016; 2016. http://dx.doi.org/10.1155/2016/2492107, ID 2492107).

Studied groups	BGL (mg/dl)	AI	AAI (%)	Insulin (μIU/ml)	HOMA-IR (mg/dl)	HOMA-β (%)
Control	75.53 ± 1.22	1.70 ± 0.06	142.63 ± 20.08	11.59 ± 1.76	2.16 ± 0.89	33.299 ± 0.908
DMSO	116.07 ± 2.66***	1.42 ± 0.04	138.58 ± 2.25	45.00 ± 0.68***	12.90 ± 0.76***	30.526 ± 1.301**
Diabetic	581.31±36.31***	16.82 ± 1.25***	6.32 ± 0.56***	101.59 ± 5.78***	145.82 ± 1.43***	7.056 ± 0.512***
NSO	95.70 ± 1.89***	1.15 ± 0.30	676.37 ± 37.56***	41.10 ± 1.87***	9.71 ± 1.25***	45.248 ± 0.456***
MET	56.37 ± 0.61***	1.73 ± 0.22	137.40 ± 12.45	30.34 ± 1.26***	4.22 ± 0.67*	-164.742 ± 0.636***
GLI	57.62 ± 1.46***	1.71 ± 0.09	141.03 ± 9.08	31.77 ± 1.42***	4.52 ± 0.45*	-212.587 ± 0.123***
NSO-MET	101.67 ± 3.05***	1.56 ± 0.36	179.13 ± 15.06*	37.73 ± 0.60***	9.47 ± 0.83**	35.125 ± 1.086**
NSO-GLI	110.16 ± 1.84***	1.65 ± 0.19	153.55 ± 10.12	36.44 ± 0.75***	9.91 ± 1.26**	27.817 ± 0.978***
Diabetic, NSO	142.76 ± 16.94**	1.00 ± 0.06	23147.62 ± 42.96***	127.86 ± 1.27***	45.07 ± 0.66***	57.710 ± 0.805***
Diabetic, MET	265.23 ± 20.67***	5.72 ± 1.45*	21.18 ± 1.08***	54.03 ± 2.45***	35.42 ± 0.56***	9.618 ± 0.526***
Diabetic, GLI	359.94 ± 17.29***	3.78 ± 1.26*	35.96 ± 8.45***	74.47 ± 5.39***	66.18 ± 1.47***	9.028 ± 0.538***
Diabetic, NSO-MET	112.46 ± 3.94***	1.42 ± 0.05	240.83 ± 16.85***	90.80 ± 2.70***	25.21 ± 0.98***	66.09 ± 1.123***
Diabetic, NSO-GLI	106.62 ± 3.79***	1.41 ± 0.89	245.85 ± 8.03***	76.11 ± 0.86***	20.04 ± 0.89***	62.814 ± 0.907***
IOMe 2	124.59 ± 3.26***	1.73 ± 0.30	137.89 ± 6.02	42.80 ± 1.18***	13.17 ± 0.45***	25.017 ± 0.625***
Diabetic, IOMe 2	224.62 ± 4.29***	12.54 ± 2.85***	8.67 ± 1.25***	63.54 ± 3.38***	35.24 ± 0.47***	14.153 ± 0.925***
IOMe 2-NSO	94.26 ± 1.43***	1.42 ± 0.09	236.49 ± 4.56***	5.49 ± 2.16***	1.28 ± 0.56	6.322 ± 0.347***
Diabetic, IOMe 2-NSO	113.54 ± 1.83***	1.52 ± 0.35	191.68 ± 8.47**	62.56 ± 1.59***	17.54 ± 1.49***	44.562 ± 0.908***
